# Supplementary figures and images for: Reactive focal drug administration associated with decreased malaria transmission in an elimination setting: Serological evidence from the cluster-randomized CoRE study
Source: PLOS Glob Public Health. 2022 Dec 5;2(12):e0001295. doi: 10.1371/journal.pgph.0001295 (PMC10021141; doi:10.1371/journal.pgph.0001295)

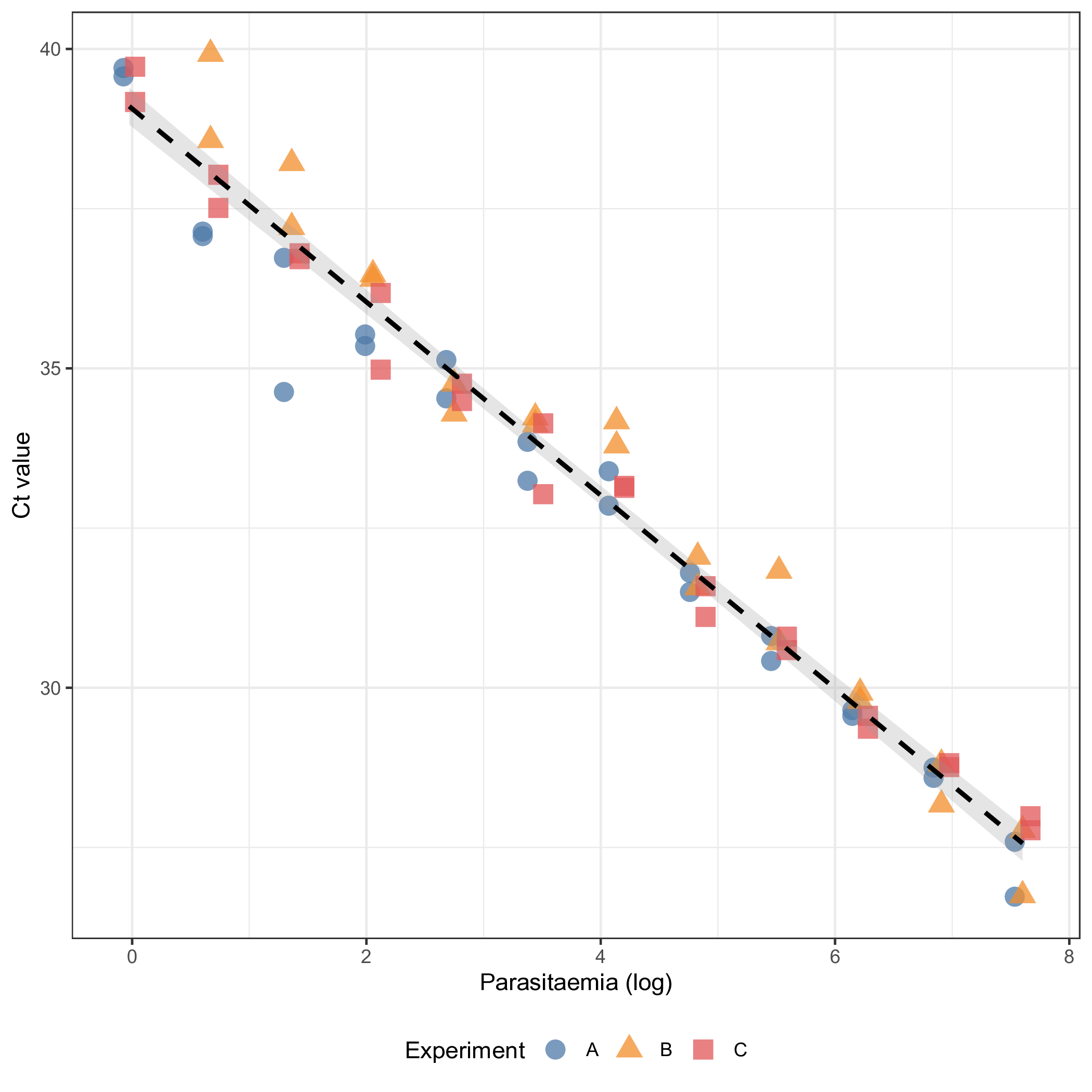

Supplement: S1 Fig — The assay was performed in duplicate (n = 3). (TIF) [file pgph.0001295.s002.tif]

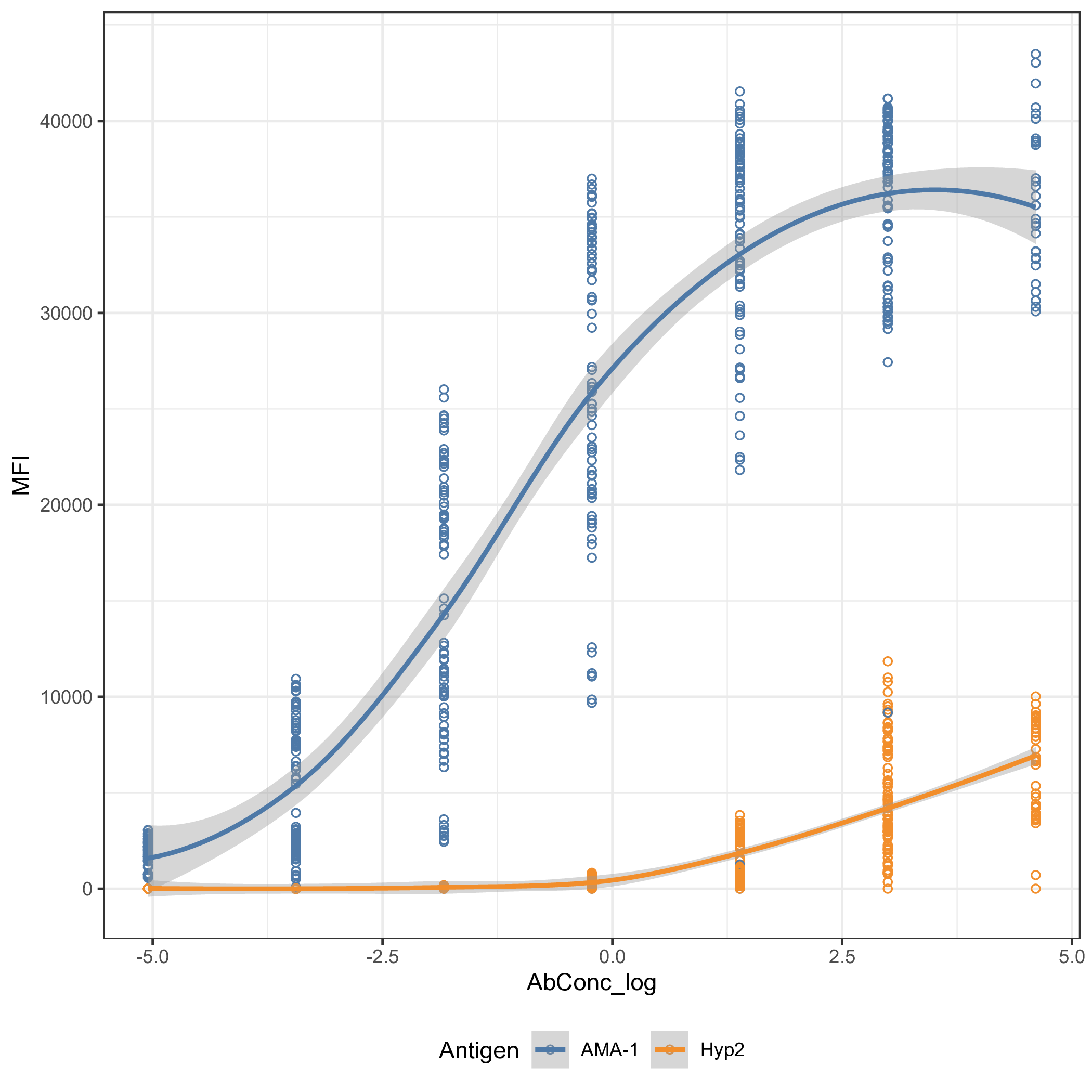

Supplement: S2 Fig — (TIF) [file pgph.0001295.s003.tif]

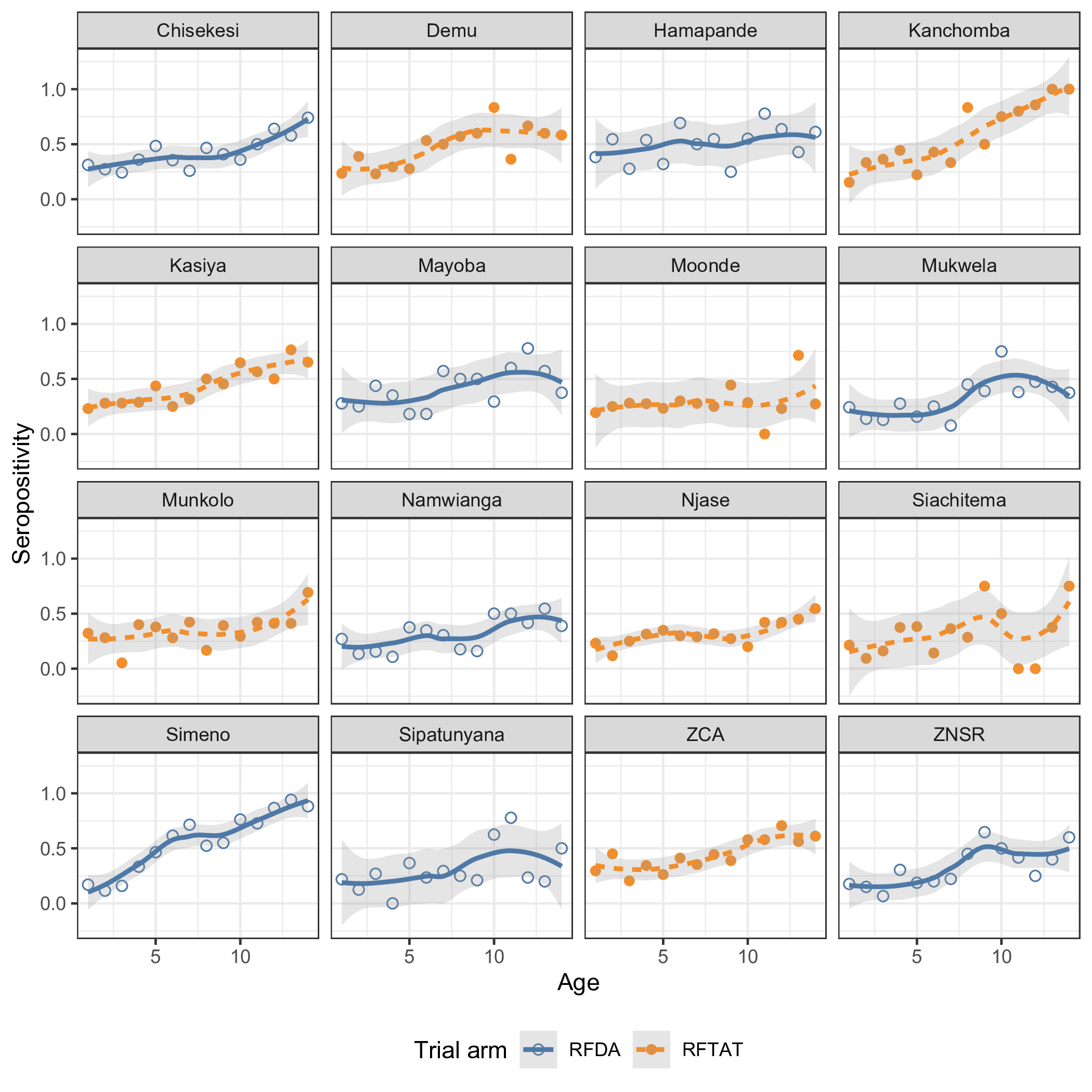

Supplement: S3 Fig — Data are fitted using a loess smoother function and 95% confidence intervals (grey shaded area). RFTAT control (black) and RFDA intervention (red) arms are shown accordingly. (TIF) [file pgph.0001295.s004.tif]

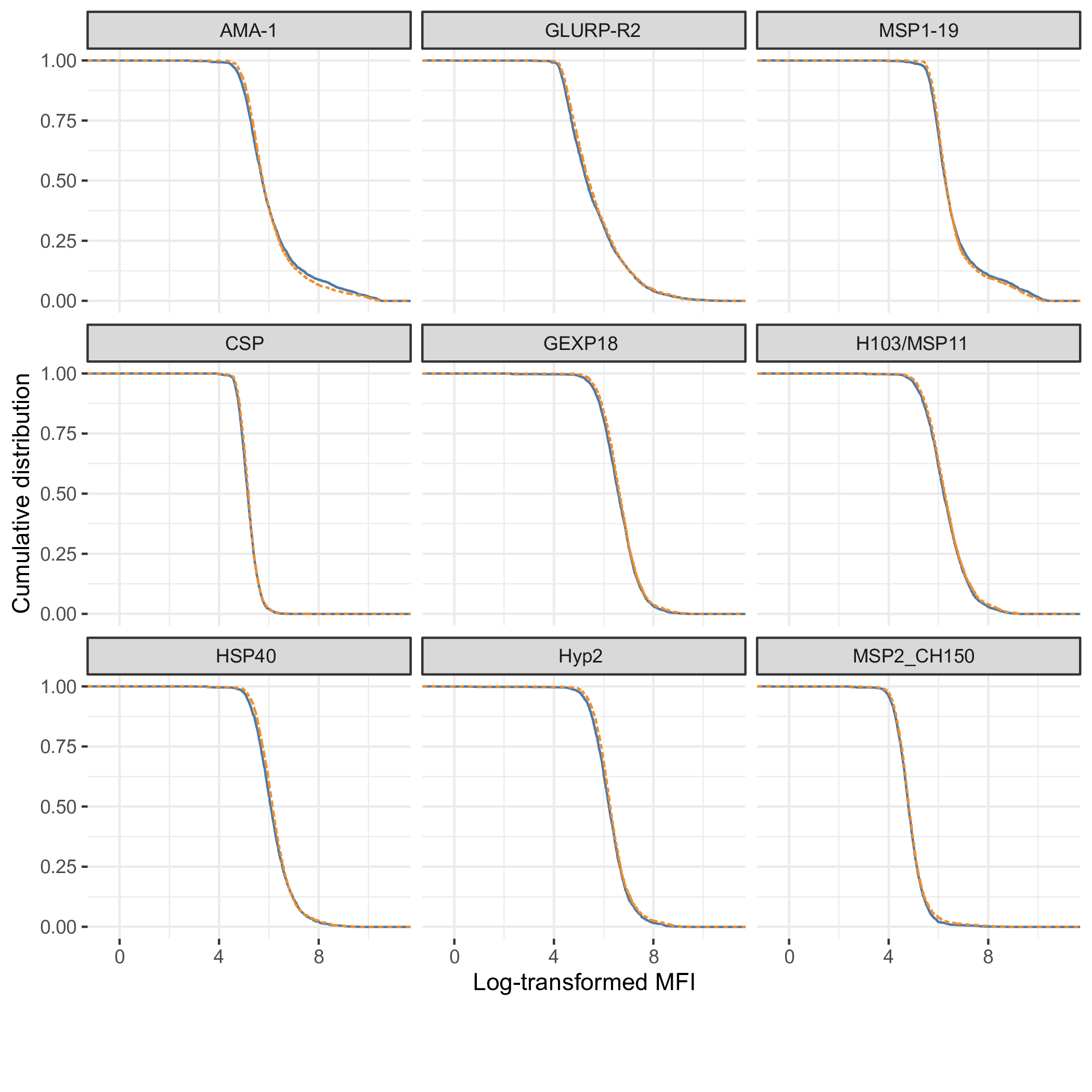

Supplement: S4 Fig — RFTAT control (orange) and RFDA intervention (blue) arms are shown accordingly. (TIF) [file pgph.0001295.s005.tif]
