# Supplementary material for: Reactive focal drug administration associated with decreased malaria transmission in an elimination setting: Serological evidence from the cluster-randomized CoRE study
Source: PLOS Glob Public Health. 2022 Dec 5;2(12):e0001295. doi: 10.1371/journal.pgph.0001295 (PMC10021141; doi:10.1371/journal.pgph.0001295)
Supplement: S1 Table — (DOCX) [file pgph.0001295.s006.docx]

| Antigen | Classification of antibody duration | FMM cutoff  (log-MFI) | # Positive / total samples (Positivity %) |
| --- | --- | --- | --- |
| AMA-1 | Long | 7.54 | 696 / 5,405 (12.9%) |
| GLURP-R2 | Long | 6.18 | 1,417 / 3,815 (37.1%) |
| MSP1-19 | Long | 6.67 | 699 / 5,253 (13.3%) |
| CSP | Short | 6.52 | 14 / 6,091 (0.2%) |
| GEXP18 | Short | 9.39 | 15 / 6,081 (0.2%) |
| Hyp2 | Short | 8.58 | 58 / 6,039 (1.0%) |
| MSP2_CH150 | Short | 6.68 | 72 / 5,188 (1.4%) |
| HSP40 Ag1 | Short | 8.19 | 85 / 5,133 (1.7%) |
| SEA-1 | Short | 8.43 | 549 / 5,544 (9.9%) |
