# Supplementary material for: Reactive focal drug administration associated with decreased malaria transmission in an elimination setting: Serological evidence from the cluster-randomized CoRE study
Source: PLOS Glob Public Health. 2022 Dec 5;2(12):e0001295. doi: 10.1371/journal.pgph.0001295 (PMC10021141; doi:10.1371/journal.pgph.0001295)
Supplement: S2 Table — (DOCX) [file pgph.0001295.s007.docx]

| Long-term antigen analysis | | | |
| --- | --- | --- | --- |
| Antigen removed | Coefficient | Standard error | P-value |
| Full analysis | -0.211 | 0.088 | 0.016 |
| AMA-1 removed | -0.205 | 0.096 | 0.033 |
| GLURP-R2 removed | -0.176 | 0.166 | 0.288 |
| MSP1-19 removed | -0.175 | 0.090 | 0.052 |
| Short-term antigen analysis | | | |
| Full analysis | -0.458 | 0.223 | 0.042 |
| MSP2_CH150 removed | -0.458 | 0.223 | 0.042 |
| H103/MSP11 removed | -0.369 | 0.267 | 0.167 |
| HSP40 Ag1 removed | -0.669 | 0.209 | 0.001 |
| Hyp2 removed | -0.456 | 0.234 | 0.051 |
| GEXP18 removed | -0.446 | 0.224 | 0.047 |
| CSP removed | -0.459 | 0.236 | 0.051 |
| Facility removed for long-term antigen analysis | | | |
| Facility removed | Interaction coefficient | Standard error | P-value |
| Chisekesi RHC | -0.214 | 0.094 | 0.023 |
| Demu Health Post | -0.225 | 0.090 | 0.012 |
| Hamapande RHC | -0.281 | 0.095 | 0.003 |
| Kanchomba RHC | -0.225 | 0.090 | 0.012 |
| Kasiya RHC | -0.236 | 0.092 | 0.011 |
| Mayoba RHC | -0.251 | 0.093 | 0.007 |
| Moonde Health Post | -0.176 | 0.090 | 0.051 |
| Mukwela RHC | -0.228 | 0.092 | 0.013 |
| Munkolo RHC | -0.188 | 0.091 | 0.039 |
| Namwianga UHC | -0.207 | 0.091 | 0.023 |
| Njase RHC | -0.193 | 0.091 | 0.033 |
| Siachitema RHC | -0.239 | 0.89 | 0.007 |
| Simeno Health Post | -0.111 | 0.090 | 0.218 |
| Sipatunyana RHC | -0.200 | 0.090 | 0.025 |
| ZCA RHC | -0.202 | 0.093 | 0.029 |
| Zambia National Service RHC | -0.195 | 0.089 | 0.028 |
| Facility removed for short-term antigen analysis | | | |
| Chisekesi RHC | -0.351 | 0.227 | 0.122 |
| Demu Health Post | -0.451 | 0.241 | 0.061 |
| Hamapande RHC | -0.429 | 0.241 | 0.074 |
| Kanchomba RHC | -0.455 | 0.239 | 0.058 |
| Kasiya RHC | -0.331 | 0.266 | 0.213 |
| Mayoba RHC | -0.498 | 0.254 | 0.050 |
| Moonde Health Post | -0.561 | 0.242 | 0.021 |
| Mukwela RHC | -0.442 | 0.251 | 0.078 |
| Munkolo RHC | -0.454 | 0.254 | 0.074 |
| Namwianga UHC | -0.444 | 0.242 | 0.067 |
| Njase RHC | -0.465 | 0.247 | 0.060 |
| Siachitema RHC | -0.494 | 0.242 | 0.041 |
| Simeno Health Post | -0.811 | 0.134 | < 0.001 |
| Sipatunyana RHC | -0.403 | 0.232 | 0.082 |
| ZCA RHC | -0.440 | 0.260 | 0.091 |
| Zambia National Service RHC | -0.417 | 0.242 | 0.085 |
