# Supplementary material for: Reactive focal drug administration associated with decreased malaria transmission in an elimination setting: Serological evidence from the cluster-randomized CoRE study
Source: PLOS Glob Public Health. 2022 Dec 5;2(12):e0001295. doi: 10.1371/journal.pgph.0001295 (PMC10021141; doi:10.1371/journal.pgph.0001295)
Supplement: S3 Table — (DOCX) [file pgph.0001295.s008.docx]

| Antigen | Seroprevalence (95% CI) | | |
| --- | --- | --- | --- |
|  | Overall | RFTAT | RFDA |
| Long-term | | | |
| AMA-1 | 11.4% (10.6 – 12.2) | 10.4% (9.3 – 11.4) | 12.4% (11.3 – 13.6) |
| GLURP-R2 | 27.1% (25.9 – 28.3) | 27.8% (26 – 29.6) | 26.4% (24.7 – 28.0) |
| MSP1-19 | 11.7% (10.9 – 12.6) | 11.2% (10.0 – 12.3) | 12.3% (11.1 – 13.5) |
| Short-term | | | |
| GEXP18 | 0.2% (0.1 – 0.4) | 0.2% (0.0 – 0.3) | 0.3% (0.1 – 0.5) |
| H103/MSP11 | 1.2% (0.9 – 1.4) | 1.4% (1.0 – 1.8) | 0.9% (0.6 – 1.2) |
| HSP40 Ag1 | 1.6% (1.3 – 2.0) | 2.0% (1.4 – 2.6) | 1.3% (0.9 – 1.7) |
| Hyp2 | 1.0% (0.7 – 1.2) | 1.3% (0.9 – 1.7) | 0.6% (0.3 – 0.9) |
| MSP2_CH150 | 1.4% (1.1 – 1.7) | 1.6% (1.1 – 2.1) | 1.1% (0.8 – 1.5) |
| CSP | 0.2% (0.1 – 0.3) | 0.3% (0.1 – 0.5) | 0.1% (0.0 – 0.3) |
