# Supplementary material for: Reactive focal drug administration associated with decreased malaria transmission in an elimination setting: Serological evidence from the cluster-randomized CoRE study
Source: PLOS Glob Public Health. 2022 Dec 5;2(12):e0001295. doi: 10.1371/journal.pgph.0001295 (PMC10021141; doi:10.1371/journal.pgph.0001295)
Supplement: S4 Table — (DOCX) [file pgph.0001295.s009.docx]

| Arm | Day | | | | |
| --- | --- | --- | --- | --- | --- |
|  | 0 | 30 | | 90 | |
|  | PCR | PCR | Loss to follow-up | PCR | Loss to follow-up |
| RFTAT (Control) | 72/608 (11.8%) | 5/463 (1.1%) | 145/608 (23.8%) | 1/390 (0.3%) | 218/608 (35.8%) |
| RFDA (Intervention) | 85/1118 (7.6%) | 2/859 (0.2%) | 259/1118 (23.1%) | 4/724 (0.6%) | 394/1118 (35.2%) |
