# Supplementary material for: Reactive focal drug administration associated with decreased malaria transmission in an elimination setting: Serological evidence from the cluster-randomized CoRE study
Source: PLOS Glob Public Health. 2022 Dec 5;2(12):e0001295. doi: 10.1371/journal.pgph.0001295 (PMC10021141; doi:10.1371/journal.pgph.0001295)
Supplement: S5 Table — (DOCX) [file pgph.0001295.s010.docx]

|  | **RFTAT (control)** | | **RFDA (intervention)** | |
| --- | --- | --- | --- | --- |
| Age | Female | Male | Female | Male |
| < 10 years | 120 | 118 | 240 | 239 |
| 10 – 19 years | 91 | 90 | 151 | 140 |
| 20 – 29 years | 41 | 34 | 80 | 62 |
| 30 – 39 years | 40 | 24 | 72 | 40 |
| 40 – 49 years | 21 | 20 | 45 | 40 |
| 50 – 59 years | 14 | 11 | 21 | 14 |
| 60 years or older | 6 | 8 | 30 | 18 |
