# Supplementary material for: Reactive focal drug administration associated with decreased malaria transmission in an elimination setting: Serological evidence from the cluster-randomized CoRE study
Source: PLOS Glob Public Health. 2022 Dec 5;2(12):e0001295. doi: 10.1371/journal.pgph.0001295 (PMC10021141; doi:10.1371/journal.pgph.0001295)
Supplement: S6 Table — (DOCX) [file pgph.0001295.s011.docx]

|  | **RFTAT (control)** | | **RFDA (intervention)** | |
| --- | --- | --- | --- | --- |
| Age | Female | Male | Female | Male |
| < 5 years | 766 | 704 | 735 | 699 |
| 5-9 years | 500 | 466 | 495 | 497 |
| >= 10 years | 376 | 313 | 408 | 317 |
