# Supplementary material for: Reactive focal drug administration associated with decreased malaria transmission in an elimination setting: Serological evidence from the cluster-randomized CoRE study
Source: PLOS Glob Public Health. 2022 Dec 5;2(12):e0001295. doi: 10.1371/journal.pgph.0001295 (PMC10021141; doi:10.1371/journal.pgph.0001295)
Supplement: S1 File — (DOCX) [file pgph.0001295.s012.docx]

Equation 1

$$y_{ij}|\pi_{ij}\sim Binomial(1,\pi_{ij})$$

$$log(\pi_{ij})=\beta I_{ij}\times\beta A_{ij}+\beta H_{ij}+\beta C_{ij}$$

$$\zeta_{j}\sim N(0,\psi)$$

where $\pi$*_ij_* is a dichotomous outcome for person *i* in health facility catchment *j*, *I_ij_* is whether the catchment was assigned to the intervention or control group, *A_ij_* is whether the child is under 5 years of age or not, *H_ij_* is a vector of household characteristics, and *C_ij_* is a vector of child characteristics.

Equation 2

$$y_{ij}|\pi_{ij}\sim Binomial(1,\pi_{ij})$$

$$log(\pi_{ij})=\beta I_{ij}+\beta H_{ij}+\beta C_{ij}$$

$$\zeta_{j}\sim N(0,\psi)$$

where $\pi$*_ij_* is a dichotomous outcome for person *i* in health facility catchment *j*, *I_ij_* is whether the catchment was assigned to the intervention or control group, *A_ij_* is whether the child is under 5 years of age or not, *H_ij_* is a vector of household characteristics, and *C_ij_* is a vector of child characteristics.

Equation 3

$$PR\left( y_{ij} | x_{ij},\delta_{i} \right)=\beta I_{i}+\beta T_{ij}+\beta I_{i}\times\beta T_{ij}+\beta D_{ij}+\beta E_{ij}+\beta S_{ij}$$

$$\zeta_{j}\sim N(0,\psi)$$

where *y_ij_* is the number of PCR-confirmed malaria infections found in health facility *i* in month *j*, *x_ij_* is the catchment population in health facility *i* and month *j*, *I_ij_* is the measure of whether the facility was included in the trial or not, *T_ij_* is a vector of time, *I_i_*$\times$*T_ij_* is the interaction of being in the trial or not and time, *D_ij_* is a vector of diagnostic tests conducted, *E_ij_* is a vector of environmental characteristics, and *S_ij_* is a vector of seasonality.
