# Supplementary material for: Reactive focal drug administration associated with decreased malaria transmission in an elimination setting: Serological evidence from the cluster-randomized CoRE study
Source: PLOS Glob Public Health. 2022 Dec 5;2(12):e0001295. doi: 10.1371/journal.pgph.0001295 (PMC10021141; doi:10.1371/journal.pgph.0001295)
Supplement: S1 Protocol — (DOCX) [file pgph.0001295.s014.docx]

Protocol:

Community-led Responses for Elimination (CoRE): A cluster randomized controlled trial of reactive case detection versus reactive drug administration in malaria elimination areas

*23^rd^ July 2019*

*Version 11*

| **Principal Investigators** |  |
| --- | --- |
| Daniel Bridges, PhD  PATH-MACEPA  National Malaria Control Centre  Chainama Hospital College Grounds  Lusaka, Zambia  Phone: +260 965273474  Email: [dbridges@path.org](mailto:dbridges@path.org) | John Miller, PhD, MPH, MA  PATH-MACEPA  National Malaria Control Centre  Chainama Hospital College Grounds  Lusaka, Zambia  Phone: +26 0977 510 414  Email: [jmiller@path.org](mailto:jmiller@path.org) |
| Captain (Dr.) Victor Chalwe, MD, MSc Medical Epidemiologist  Disease Control Specialist  Ministry of Health  Province Medical Office  PO BOX 710010  Mansa  Luapula Province  Tel: +260-979-883237  Email: [victorchalwe2011@yahoo.com](mailto:victorchalwe2011@yahoo.com) |  |
| **Co- investigators** |  |
| David Larsen, PhD, MPH  Syracuse University  Department of Public Health, Food Studies & Nutrition  Syracuse, NY 13244  Phone: +1 315 443-4059  Email: [dalarsen@syr.edu](mailto:%20dalarsen@syr.edu) | Kafula Silumbe  PATH-MACEPA  National Malaria Control Centre Chainama Hospital College Grounds Lusaka, Zambia  Phone: +260 978774230 Email: [ksilumbe@path.org](mailto:ksilumbe@path.org) |
| Hawela Moonga, MS  National Malaria Control Center  Zambia Ministry of Health  Chainama Hospital  Phone: +26 0977 659 082  Email: [hawela@yahoo.co.uk](mailto:hawela@yahoo.co.uk) | Busiku Hamainza, MPH  National Malaria Control Center  Zambia Ministry of Health  Chainama Hospital  Phone: +26 0977 941761  Email: [bossbusk@gmail.com](mailto:bossbusk@gmail.com) |
| Smita Das  PATH  2201 Westlake Ave. Suite 200  Seattle, Washington 98121  Phone: +1 206 302 4713  Email: [sdas@path.org](mailto:sdas@path.org) | Gonzalo Domingo  PATH  2201 Westlake Ave. Suite 200  Seattle, Washington 98121  Phone: +1 206 302 4741  Email: gdomingo@path.org |
| Jade Benjamin-Chung  Division of Epidemiology & Biostatistics  University of California, Berkeley  2121 Berkeley Way Rm 5302  Berkeley, CA 94720-7360  Email: [jadebc@berkeley.edu](mailto:jadebc@berkeley.edu) |  |
|  |  |
|  |  |

# Table of Contents

[Table of Contents 3](#_Toc508023711)

[Abbreviations 5](#_Toc508023712)

[Definitions of Key Terminology 5](#_Toc508023713)

[Health Facility hierarchy in Zambia 6](#_Toc508023714)

[Project summary 7](#_Toc508023715)

[1 Background 9](#_Toc508023716)

[1.1 Introduction 9](#_Toc508023717)

[1.2 Mass drug administration (MDA) 10](#_Toc508023718)

[1.3 Rationale for RFDA 10](#_Toc508023719)

[1.4 Documenting zero transmission 11](#_Toc508023720)

[1.5 High sensitivity Alere™ Malaria P.f RDT (HS RDT) 12](#_Toc508023721)

[1.6 Goal 14](#_Toc508023722)

[1.7 Aims 14](#_Toc508023723)

[2 Research Plan 14](#_Toc508023724)

[2.1 Study Design 14](#_Toc508023725)

[2.2 Treatment regimes 16](#_Toc508023726)

[2.2.1 Artemether-lumefantrine (AL) – Control arm 16](#_Toc508023727)

[2.2.2 Dihydroartemisinin - piperaquine (DHAP) 17](#_Toc508023728)

[2.3 Study participants 18](#_Toc508023729)

[2.4 Reactive responses 19](#_Toc508023730)

[2.4.1 Research Response 20](#_Toc508023731)

[2.4.2 Directly Observed Treatment (DOT) 23](#_Toc508023732)

[2.5 Surveys 23](#_Toc508023733)

[2.6 Routine Data Reporting 24](#_Toc508023734)

[2.7 Consent 24](#_Toc508023735)

[2.7.1 Subject capacity 25](#_Toc508023736)

[2.8 Research Strategy by Aim 25](#_Toc508023737)

[2.8.1 Primary Aim 25](#_Toc508023738)

[2.8.2 Secondary Aim 1 26](#_Toc508023739)

[2.8.3 Secondary Aim 2 26](#_Toc508023740)

[2.8.4 Secondary Aim 3 27](#_Toc508023741)

[2.8.5 Secondary Aim 4 28](#_Toc508023742)

[2.8.6 Secondary Aim 5 29](#_Toc508023743)

[2.8.7 Secondary Aim 6 30](#_Toc508023744)

[2.8.8 Secondary Aim 7 31](#_Toc508023745)

[2.9 Survey Sample Size 31](#_Toc508023746)

[2.10 Study Logistics 32](#_Toc508023747)

[2.10.1 Community Mobilization and Sensitization Activities 32](#_Toc508023748)

[2.10.2 Incentives 33](#_Toc508023749)

[2.11 Data Management 33](#_Toc508023750)

[2.12 Personnel 33](#_Toc508023751)

[2.12.1 Survey team 33](#_Toc508023752)

[2.12.2 Standard CHW Response (RFTAT / RFDA) 33](#_Toc508023753)

[2.12.3 Research Response (RFTAT / RFDA) 33](#_Toc508023754)

[2.13 Sample Analysis Assays 34](#_Toc508023755)

[2.13.1 PCR analysis for presence of Plasmodium falciparum 34](#_Toc508023756)

[2.13.2 Genotype analysis on positive samples 34](#_Toc508023757)

[2.13.3 Serology 34](#_Toc508023758)

[2.14 Challenges and Limitations 34](#_Toc508023759)

[2.14.1 False Positive Test Results 34](#_Toc508023760)

[2.14.2 Adequate RDT and ACT Supplies 35](#_Toc508023761)

[3 Ethical Issues 36](#_Toc508023762)

[3.1 Adequacy of protection against risks 36](#_Toc508023763)

[3.2 Data and safety monitoring plan 39](#_Toc508023764)

[3.3 Potential benefits of the proposed research to the participants and others 39](#_Toc508023765)

[3.4 Collection of specimens 40](#_Toc508023766)

[3.5 Protocol Accessibility 40](#_Toc508023767)

[4 Timeline 41](#_Toc508023768)

[5 References 42](#_Toc508023769)

[6 Appendices 45](#_Toc508023770)

# Abbreviations

ACT Artemisinin Combination Therapy

AE Adverse Event

AL Artemether–Lumefantrine

CHW Community health worker

DBS Dried Blood Spot

DHA Dihydroartemisinin

DHAP Dihydroartemisinin – Piperaquine

DSMB Drug Safety Monitoring Board

HC Health Centre (see RHC)

HP Health Post (see RHP)

HS RDT High Sensitivity Alere™ Malaria Ag P.f RDT

IRS Indoor-residual spraying

LOD Limit of Detection

LLIN Long Lasting Insecticide treated mosquito Net

NMCC National Malaria Control Center

RDT Rapid Diagnostic Test

PCD Passive Case Detection

PCR Polymerase Chain Reaction

RFDA Reactive Focal Drug Administration

RFTAT Reactive Test and Treat

RHC Rural Health Centre

RHP Rural Health Post

SP Sulphadoxine-Pyrimethamine

SAE Serious Adverse Event

WHO World Health Organization

# Definitions of Key Terminology

PCD Passive Case Detection – Symptomatic individuals present to a health center or health post and are tested for malaria with a rapid diagnostic test (RDT) or microscopy, and treated with an appropriate antimalarial if positive.

RFDA Reactive Focal Drug Administration – A reactive response consisting of treating all individuals within a defined radius of each RDT-confirmed incident malaria case with dihydroartemisinin-piperaquine (DHAP).

RFTAT Reactive Focal Test and Treat – A reactive response consisting of testing all individuals within a defined radius of each RDT-confirmed incident malaria case with an RDT and treating all positive individuals with artemether-lumefantrine (AL).

CHW Responses The majority of RFTAT or RFDA responses will be exclusively performed by a single CHW operating similarly to standard reactive case detection procedures in rural areas.

Research Responses These responses will augment the CHW response, with a sample collector, enumerator and supervisor. This team will also make repeat visits to follow individuals enrolled in a reactive response longitudinally.

# Health Facility hierarchy in Zambia

To avoid confusion, it is necessary to clarify terminology around health facilities. For the purposes of this document, the term rural health clinic (HC / RHC) will refer to all government run facilities that act as the primary location for health care service delivery and which are manned by professional health workers. In contrast, a rural health post (HP / RHP) is a satellite facility attached to an individual health centre that aims to expand health care access for a few key interventions, namely malaria testing and treatment. HP’s are predominantly manned by volunteer community health workers. The term health facility will refer to both HC’s and HP’s.

# Project summary

| **Primary Aim** | Compare the effectiveness of RFDA using DHAP with RFTAT using AL in achieving zero seropositivity in children under fifteen |
| --- | --- |
| **Secondary Aims** | 1. Compare the effectiveness of RFDA using DHAP with RFTAT using AL in reducing RDT confirmed malaria incidence through passive case detection at health facilities. 2. Compare the effectiveness of RFDA using DHAP with RFTAT using AL in reducing the prevalence of malaria and preventing re-infection in individuals receiving reactive responses. 3. Compare the cost-effectiveness of RFDA using DHAP with RFTAT using AL in reducing the burden of malaria in the community. 4. Measure the proportion of *P. falciparum* infections likely attributable to importation and local transmission using parasite genotyping as well as defining genotype spatial distribution. 5. Assess the utility of using serology to measure short term changes in malaria transmission and evaluate malaria elimination programs 6. Assess the feasibility of using remotely sensed malaria risk maps to identify areas with higher potential for local malaria transmission. 7. Assess the utility of a HS RDT in identifying additional standard RDT sub-patent infections in the RFDA arm |
| **Study Site & Target Populations** | Population of ~130,000 people in ~30,000 households in 16 Health Center catchment areas in Southern Province. |
| **Study design** | Cluster randomized controlled trial will be used to evaluate the impact of RFDA intervention against current standard of care for the impact on seropositivity in children under fifteen years, passive surveillance for confirmed malaria case incidence, and elimination of transmission from hotspots |
| **Primary outcome measures for assessing program impact** | 1. Malaria seropositivity in children under fifteen after two-year intervention within health center catchment areas 2. Confirmed malaria incidence as measured through routine passive case detection at health centers and health posts throughout the selected health center catchment areas |
| **Primary data collection & sampling method** | Sampling after the interventions will be used to determine malaria seropositivity in children under fifteen in each health center catchment population.  The routine government rapid reporting system will be used to determine confirmed malaria case incidence throughout the entire study area.  Longitudinal cohorts will be used to determine clearance and re-infection rates for each arm from a subset of individuals participating in reactive responses. |
| **Sample size** | - Simple random sample survey of children under fifteen in all health clinic catchments population : 8,000 - Routine reporting for malaria case incidence: ~130,000 (all ages) - Reactive responses in all areas: ~30,000 (all ages) - Re-infection / importation / serology in a subset of the study participants: ~9,000 (all ages) |
| **Statistical & analytic plan** | A difference-in-differences approach will be used to assess changes in both primary and secondary outcomes among intervention and control groups, using mixed effects logistic and Poisson models for the outcomes of parasite seropositivity and confirmed malaria incidence. Clearance and prevention of reinfection from areas receiving rapid responses will be determined through Poisson regression. Month-by-month estimates of effectiveness will be analyzed using the routine rapid reporting of confirmed malaria incidence.  We will estimate direct effects and spillover effects of the reactive, focal malaria interventions on malaria incidence and prevalence and assess whether effects vary by intervention coverage, distance to intervention, and time from incident case detection. We will then pool this data with other similar trials using fixed and random effects. |

# Background

## Introduction

The Zambia National Malaria Strategic Plan (2011-2016) calls for the creation of five malaria-free zones by 2015


^1^. Enhanced malaria surveillance has been expanded to cover a large portion of Southern Province, an area with a documented decline in malaria prevalence to <10% during Malaria Indicator Surveys (MIS) conducted in 2006, 2008, 2010, 2012 and 2015


^2–6^. The enhanced surveillance data suggest some areas within Southern Province are candidates for eliminating malaria and documenting zero transmission; however, questions remain about the strategies required to move from very low to zero transmission as well as how to document zero transmission.

Implementing and maintaining a high quality surveillance and passive case detection (PCD) system throughout the target population is critical to achieving elimination


^7^. Even with universal access to malaria diagnosis and treatment, however, passive case detection PCD will not identify asymptomatic infected individuals


^8^. Reactive case detection is a method to identify and clear these hidden reservoirs, which are often spatially clustered in a population


^9^. In areas of low transmission, incident malaria cases can be used as an indicator of local malaria transmission


^10^. A reactive response uses incident cases as the means to target a specific geographical area where the probability of additional infected individuals is higher than in the general population


^9^.

In Southern Province, a reactive focal test and treat (RFTAT) strategy to reduce foci of infection has been implemented by the National Malaria Control Program since 2012 (Appendix 1), which was adapted from an urban reactive case detection programme (Appendix 2). In rural areas this strategy consists of testing all individuals within approximately 140 meters of an incident malaria case confirmed by rapid diagnostic test (RDT) and treating all positive individuals with artemether-lumefantrine (AL) (or referring as appropriate). In the context of malaria elimination in Zambia, this strategy has been termed Step D as it comprises the community-level surveillance system that accompanies a series of other steps (Figure 1), including Step B (health center based malaria rapid reporting system) and Step C (mass screen and / or treat activities in moderate transmission zones), all of which are active in Southern Province. A number of questions remain about the Step D approach, including its sensitivity to identify residual foci of malaria transmission, the appropriate spatial scale of the reaction, the implications of using an imperfect diagnostic test to direct treatment, and the overall effectiveness of the strategy to reduce malaria transmission to zero.


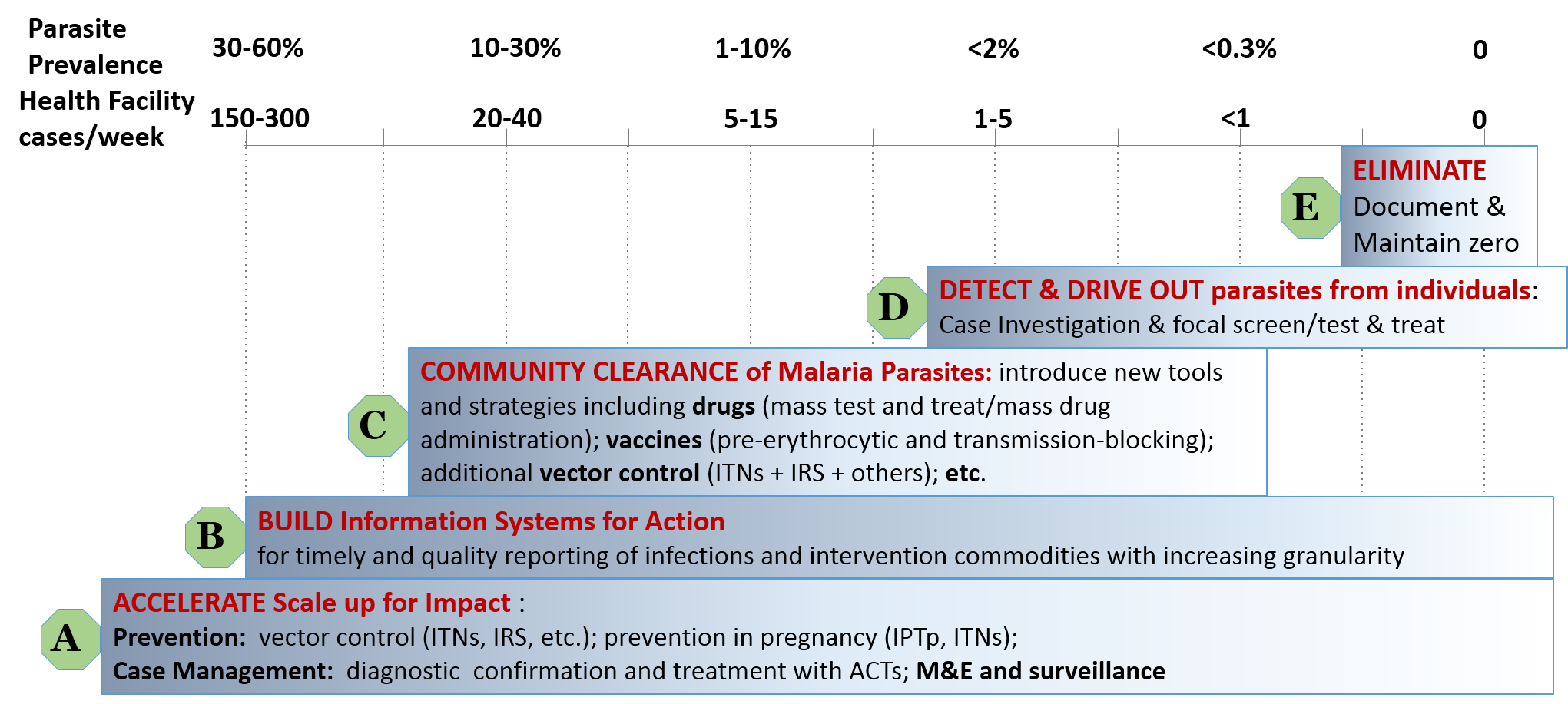


Figure 1 – Schematic highlighting the overall Malaria Control and Elimination Partnership for Africa (MACEPA) concept for malaria interventions across a range of transmission intensities.

## Mass drug administration (MDA)

MDA, as the name suggests, consists of the treatment of an entire population with a therapeutic dose for the treatment of the infectious agent, in this case *Plasmodium spp*.


^11^ Despite the notable success of MDA in eliminating malaria from island populations e.g. on Aneityum, Vanuatu,


^12^ to wide-scale implementation in China


^13^ it has fallen out of favour due to concerns over efficacy, cost, and drug resistance. Nevertheless, MDA approaches have been very successful in controlling other neglected tropical diseases and a recent Cochrane review supports the exploration of MDA as another tool in the arsenal in the quest for malaria elimination


^14^. Accordingly, as part of an invigorated approach to achieving local malaria elimination, the National Malaria Control Program with support from PATH MACEPA are evaluating community wide MDA administered through campaigns in Southern Province, Zambia (Step C, Figure 1). MDA with DHAP in conjunction with high levels of vector control have been modelled to be effective over the short term to reduce malaria parasitemia to sufficiently low levels to possibly further achieve elimination with a concomitant routine strategy. A campaign-based MDA approach can be costly and operationally challenging, and for these reasons, maintaining for a prolonged period would be inadvisable. One notable feature of historical MDA campaigns is that resurgence characteristically follows its cessation


^11,14^.

We therefore sought to develop a maximum impact protocol that was sustainable and could be applied either after the cessation of MDA or in an area of low transmission where the cost-benefit of MDA would not apply. This protocol is designed to help test alternative approaches to help optimize the current RFTAT strategy and to align with malaria elimination priorities of the Ministry of Health.

## Rationale for RFDA

The current RFTAT strategy is limited by both the sensitivity of the RDT used to detect asymptomatic infections and the limited chemoprophylaxis provided when using AL. Reactive focal drug administration (RFDA) with dihydroartemisinin-piperaquine (DHAP) could potentially reduce malaria transmission more rapidly. In low transmission settings, low-parasitaemia infections are thought to be increasingly important in sustaining malaria transmission


^15^. In areas with low malaria transmission (<20% parasite prevalence) as much as 80% of malaria infections may not be detectable through microscopy


^16^, which has a similar sensitivity to the RDTs used in the RFTAT intervention


^17^. Only treating individuals who test positive will miss a large portion of the asymptomatic malaria infections in the population. Furthermore, only treating individuals who test positive misses the opportunity to provide chemoprophylaxis to protect the local at-risk population around the incident malaria case. The lack of chemoprophylaxis is the principle reason why screening and treatment campaigns are modeled to be less effective than drug administration campaigns


^18^.

The choice of drug is crucial in providing effective chemoprophylaxis to the at-risk population. Artemisinins have short half-lives that are measured in hours; partner drugs are therefore typically selected for their longer-lasting effects


^19^. The lumefantrine component of AL has a half-life of 4-6 days


^20^. In contrast, the piperaquine component of dihydroartemisinin-piperaquine (DHAP) has a half-life of 28 days or more


^21^. The extended half-life of DHAP will provide increased prophylaxis for individuals receiving RFDA and potentially reduce malaria transmission faster than RFTAT with AL or even RFDA with AL.

## Documenting zero transmission

Documenting zero transmission requires tools that are sufficiently sensitive to declare that local transmission is not occurring. Routine surveillance systems may not be able to provide this level of sensitivity. However, there are two indirect approaches to assess the success of elimination programs and document zero transmission. First, the genetic diversity of the parasites both within infected individuals and within the local human population reflects transmission patterns. As malaria transmission decreases, parasite genetic diversity decreases in the absence of importation. By defining the molecular fingerprint or barcode for each parasite, inferences can be made about the relationship between individual infections and the transmission range and dispersal capacity of the local vector population, as well as the frequency of imported malaria. Genotyping has not yet been used as an outcome to evaluate the effectiveness of malaria interventions but has the potential to be an important piece of evidence to direct elimination efforts and document zero transmission


^22^.

Second, serology can be used to document changes in malaria transmission, and, as such, has been used to confirm malaria elimination from a number of countries, e.g., Greece


^23^. Serological profiling of a target population is fundamentally different to measuring parasite prevalence as it represents the cumulative exposure to the parasite. Seroconversion to any antigen is a function of antigen exposure, antigen immunogencity i.e. its ability to provoke an immune response, and the decay rate of the antibody response. Assuming that all individuals in a population share the same exposure risk, and that the immunogenicity of different parasite strains is comparable, then the likelihood of being seropositive increases with age. By calculating the age-specific seroconversion rates at two time points, one can measure whether transmission has increased or decreased. Traditional anti-malarial antibody serology has focused on total antibody responses, e.g. using whole parasite lysates. This is an excellent approach to document zero transmission, as all immune responses to all parasite antigens are measured as an aggregate. This ensures that exposure is measured with the highest sensitivity and longest exposure history. However immune responses can persist for decades, meaning that an individual may remain seropositive for a prolonged period, even in the absence of any new challenge. This approach is therefore only appropriate for measuring cumulative long-term exposure. Shorter-term changes in exposure can be measured by focusing on specific malaria antigens that have shorter half-lives or are less immunogenic


^24,25^.

The entomological inoculation rate is the best measure of malaria transmission intensity as it directly measures transmission. However finding *Anopheles* mosquitoes in low transmission areas of Southern Province has historically proven difficult making the direct measurement of the entomological inoculation rate impossible. Instead, the entomological inoculation rate can be modeled through measuring serological responses to malaria antibodies to show long-term changes


^25^. Serological responses to anopheline salivary peptides in low transmission settings have proven a suitable surrogate marker to document heterogeneity in mosquito exposure between individuals and locations


^26^.

## High sensitivity Alere™ Malaria P.f RDT (HS RDT)

A proportion of *P. falciparum* malaria infections, particularly those that are subclinical, are subpatent. As attention shifts from identifying clinical infections to all infections, the existing diagnostics e.g. microscopy and *Pf* HRP2-based rapid diagnostic tests (RDTs), lack the sensitivity to detect asymptomatic and low density infections (Figure 2)


^27^. For example, microscopy fails to detect a large proportion of *Pf* PCR-positive individuals, up to 20% in high transmission settings and 80% in low transmission settings


^15,28^. Similarly, RDT performance is only marginally better than microscopy and varies greatly depending on the product


^28^. Both of these diagnostics have similar limits of detection of ~100-200 parasites/µL and 800 picograms/mL HRP2 (Figure 2)


^27^. Other tools such as qPCR are highly sensitive and can detect as low as 1 p/µL (Figure 2)


^27^, but lack key attributes required for large-scale field use: ease of use, low cost, short time-to-results, and portability. Thus, to improve the efficiency of active malaria infection detection tactics, new, low cost, and portable tools with improved limit of detection (LOD) are needed


^29^.


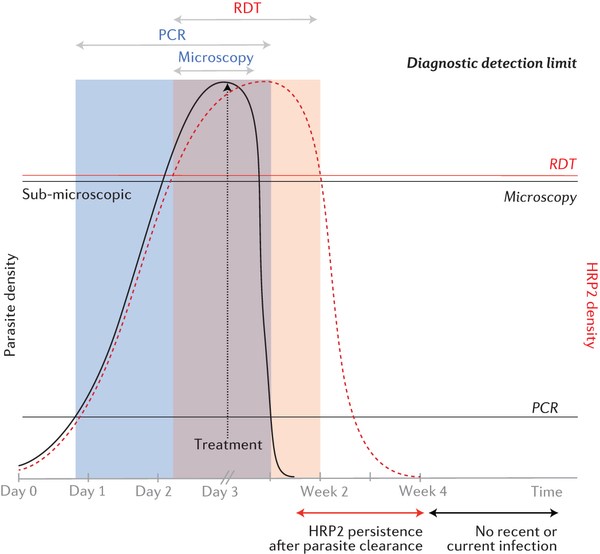


Figure 2 - Schematic of diagnostic detection limits with respect to parasite and HRP2 density


^27^. Parasite density (black curve) and HRP2 density (red curve) are shown over days prior to treatment and in weeks after treatment. Horizontal lines indicate the detection limits of respective diagnostics. The blue shaded area shows detectability of parasites by microscopy and/or PCR, whereas the red shaded area shows detectability of HRP2 by RDT.

A new generation of diagnostic tests is under development and has a significantly lower LOD. The first of these that will become available in 2017 is the high sensitivity Alere™ Malaria Ag P.f. RDT (HS RDT) for *Pf* HRP2. This test has a tenfold improvement in *Pf* HRP2 LOD from 800 pg/mL to 80 pg/mL, as demonstrated with recombinant *Pf* HRP2 and several native *Pf* HRP2 types from axenic parasite cultures. Further validation of the HS RDT with a panel of clinical specimens from asymptomatic study participants in Myanmar and Uganda show that the order of magnitude of improvement in LOD indicate (1) the ability of the test to reliably detect infections with as low as 1 p/µL as compared to the 50-200 p/µL detected by microscopy or current RDTs and (2) the detection of *Pf* HRP2 as low as 25 pg/mL as compared to 800 pg/mL by current RDTs. The results indicate a significant increase in sensitivity for *Pf* detection in low density subclinical infections with the HS RDT compared to the current commercial RDTs. Furthermore the HS RDT has the same form factor and work flow as current RDTs and therefore has negligible barriers to adoption.

While early studies of this *Pf* HRP2-based HS RDT are highly promising, it has not yet received WHO endorsement as an effective diagnostic tool in ACD strategies and clinical case management. As a result, treatment decisions based on the results from the HS RDT result are inappropriate.

## Goal

The goal of this research is to guide malaria elimination programs in Zambia on how to best utilize reactive responses to accelerate toward zero transmission and to establish, document and sustain malaria free zones.

## Aims

The study’s goal will be addressed through the following specific aims:

**Primary Aim**: Compare the effectiveness of RFDA using DHAP with RFTAT using AL in achieving zero seropositivity in children under fifteen.

**Secondary Aim 1**: Compare the effectiveness of RFDA using DHAP with RFTAT using AL in reducing RDT confirmed malaria incidence through passive case detection at health facilities.

**Secondary Aim 2**: Compare the effectiveness of RFDA using DHAP with RFTAT using AL in reducing the prevalence of malaria and preventing re-infection in individuals receiving reactive responses.

**Secondary Aim 3**: Compare the cost-effectiveness of RFDA using DHAP with RFTAT using AL in reducing the burden of malaria in the community.

**Secondary Aim 4**: Measure the proportion of *P. falciparum* infections likely attributable to importation and local transmission using parasite genotyping as well as defining genotypes spatial distribution.

**Secondary Aim 5**: Assess the utility of using serology to measure short-term changes in malaria transmission and evaluate malaria elimination programs.

**Secondary Aim 6**: Assess the feasibility of using remotely sensed malaria risk maps to identify areas with higher potential for local malaria transmission.

**Secondary Aim 7:** Assess the utility of a HS RDT in identifying additional standard RDT sub-patent infections in the RFDA arm.

# Research Plan

## Study Design

The study design will be a cluster randomized trial comparing an enhanced strategy for malaria elimination (RFDA with DHAP) with the current strategy (RFTAT with AL). Impact evaluation will consist of: 1) community-based, cross-sectional measurement of malaria seropositivity in children aged ≥ 1 month and < 15 years through a random sample survey at the end (two years) of the intervention period; 2) longitudinal follow-up of a subset of individuals participating in reactive responses by a research team; and 3) detection of incident malaria cases and total outpatient attendance at health centers and health posts.

A total of sixteen health centers will be targeted in this RCT (Table 1). The proposed areas were selected from the 11 districts within Southern Province, Zambia that have successfully implemented RFTAT surveillance with the following criteria: a mean monthly incident malaria case load of < 25 and a mean test positivity during ongoing RFTAT of at least 5%. Where multiple health centers fulfilled the inclusion criteria, preference was given to those that are geographically close to one another (minimum 5km buffer to reduce contamination) and are accessible all year round. Selected health centres will be randomized to either control or intervention arms, with 8 health centers per arm (Figure 3).

| Organisation Unit | Confirmed Cases of Malaria (Sep ’13 – Aug ’14) | | | | Positivity Rate (%) during RFTAT |
| --- | --- | --- | --- | --- | --- |
|  | Clinic | Community | Total | Average cases per month |  |
| Chisekesi Rural Health Centre | 176 | 77 | 253 | 21 | 9.4 |
| Habulile Rural Health Centre | 133 | 109 | 242 | 20 | 9.3 |
| Katombora Health Post | 123 | 78 | 201 | 17 | 12 |
| Kazungula Health Post | 71 | 20 | 91 | 8 | 7.9 |
| Keemba Rural Health Centre | 58 | 89 | 147 | 12 | 6.8 |
| Mayoba Rural Health Centre | 46 | 37 | 83 | 7 | 9.4 |
| Moonde Rural Health Clinic | 0 | 214 | 214 | 18 | 8.3 |
| Mubanga Rural Health Centre | 77 | 139 | 216 | 18 | 5.3 |
| Mukwela Rural Health Centre | 86 | 157 | 243 | 20 | 7.8 |
| Munkolo Rural Health Centre | 42 | 76 | 118 | 10 | 6.9 |
| Musokotwane Health Post | 38 | 53 | 91 | 8 | 8.2 |
| Muzya Rural Health Centre | 40 | 83 | 123 | 10 | 9.9 |
| Nadezwe Rural Health Centre | 58 | 107 | 165 | 14 | 11.2 |
| Namwianga Urban Health Centre | 56 | 140 | 196 | 16 | 5.2 |
| Ngwezi Rural Health Centre | 11 | 169 | 180 | 15 | 8.3 |
| Pangwe Rural Health Centre | 21 | 127 | 148 | 12 | 19.2 |
| Siachitema Rural Health Centre | 14 | 119 | 133 | 11 | 7.4 |
| Simooya Rural Health Centre | 142 | 40 | 182 | 15 | 8.7 |
| Zambia National Service Rural Health Centre | 78 | 40 | 118 | 10 | 10.7 |
| ZCA Rural Health Centre | 215 | 47 | 262 | 22 | 8.3 |

Table 1 – Proposed selected health centre catchment areas along with data from these facilities for the last year.

**Control Arm** - PCD at health centers and health posts, with RFTAT in response to RDT confirmed incident malaria cases as currently outlined in the Step D protocol (Appendix 1). RFTAT consists of screening all individuals within 140 meters of an incident malaria case using a RDT and treating those testing positive with AL.

**Intervention Arm** - PCD at health centers and health posts, with RFDA in response to RDT confirmed incident malaria cases. RFDA consists of treating all individuals within 140 meters of an incident malaria case with DHAP.


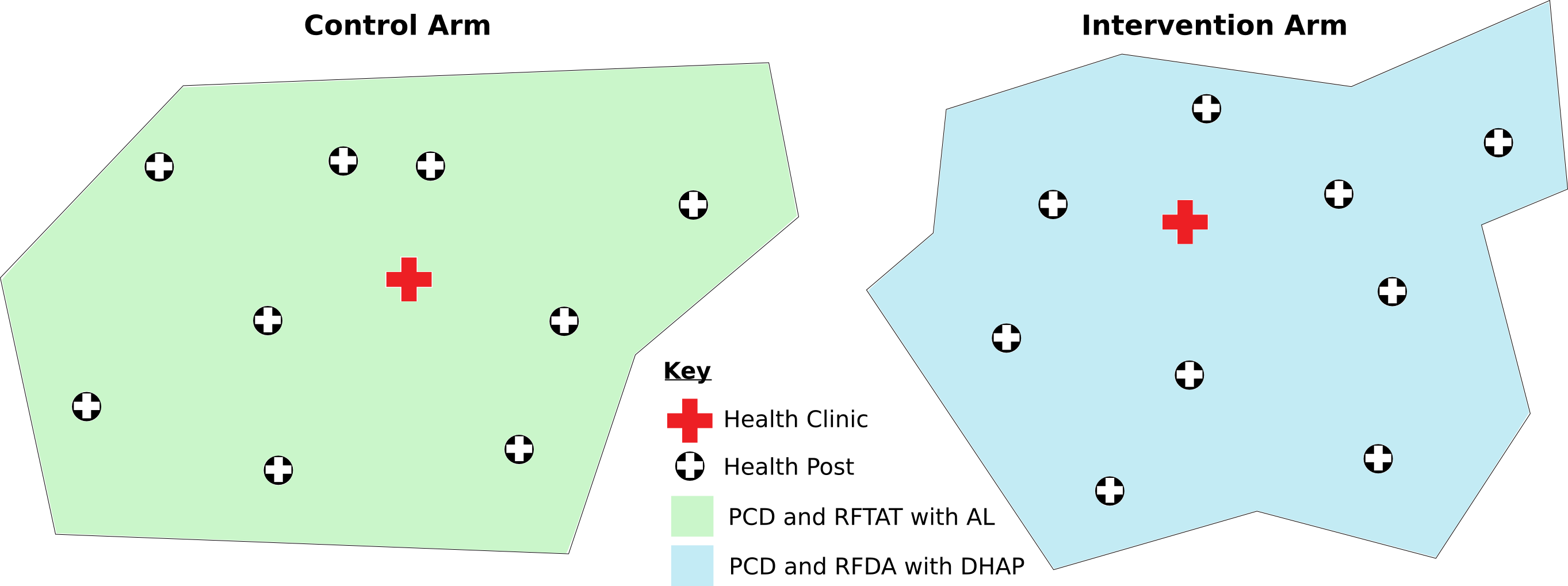


Figure 3 - Schematic showing control and intervention arms proposed at the health center catchment level. PCD, passive case detection; RFTAT, reactive focal test and treat; RFDA, reactive focal drug administration; AL, artemether-lumefantrine; DHAP, dihydroartemisinin-piperaquine.

## Treatment regimes

### Artemether-lumefantrine (AL) – Control arm

Any individual testing positive for circulating *P. falciparum* antigen by RDT will receive treatment of age- and weight-appropriate doses of AL unless below 3 months in age or 5kg in weight, as per Zambia National Malaria Treatment Guidelines. Women of reproductive age (12-49 years) will be asked about their pregnancy status and if pregnant, the last time they felt the baby move. For women reporting that they are pregnant and have not felt the baby move (still in the first trimester) they will be referred to the nearest health center if testing positive. Pregnant women beyond the first trimester who test positive will be treated with AL according to Zambia National Malaria Treatment Guidelines. If a child with a weight less than 5kg or age less than or equal to 3 months tests positive for circulating malaria antigen they will be treated with sulfadoxine-pyrimethamine (SP), as per Zambia National Malaria Treatment Guidelines, or referred to the health center as appropriate. A summary of the decision tree that will be adhered to in this study is shown in Figure 4. A fixed dose of AL currently consists of combination tablets containing 20 mg of artemether and 120 mg of lumefantrine, given twice a day for 3 days. Patients will be encouraged to take AL with food to aid drug absorption.


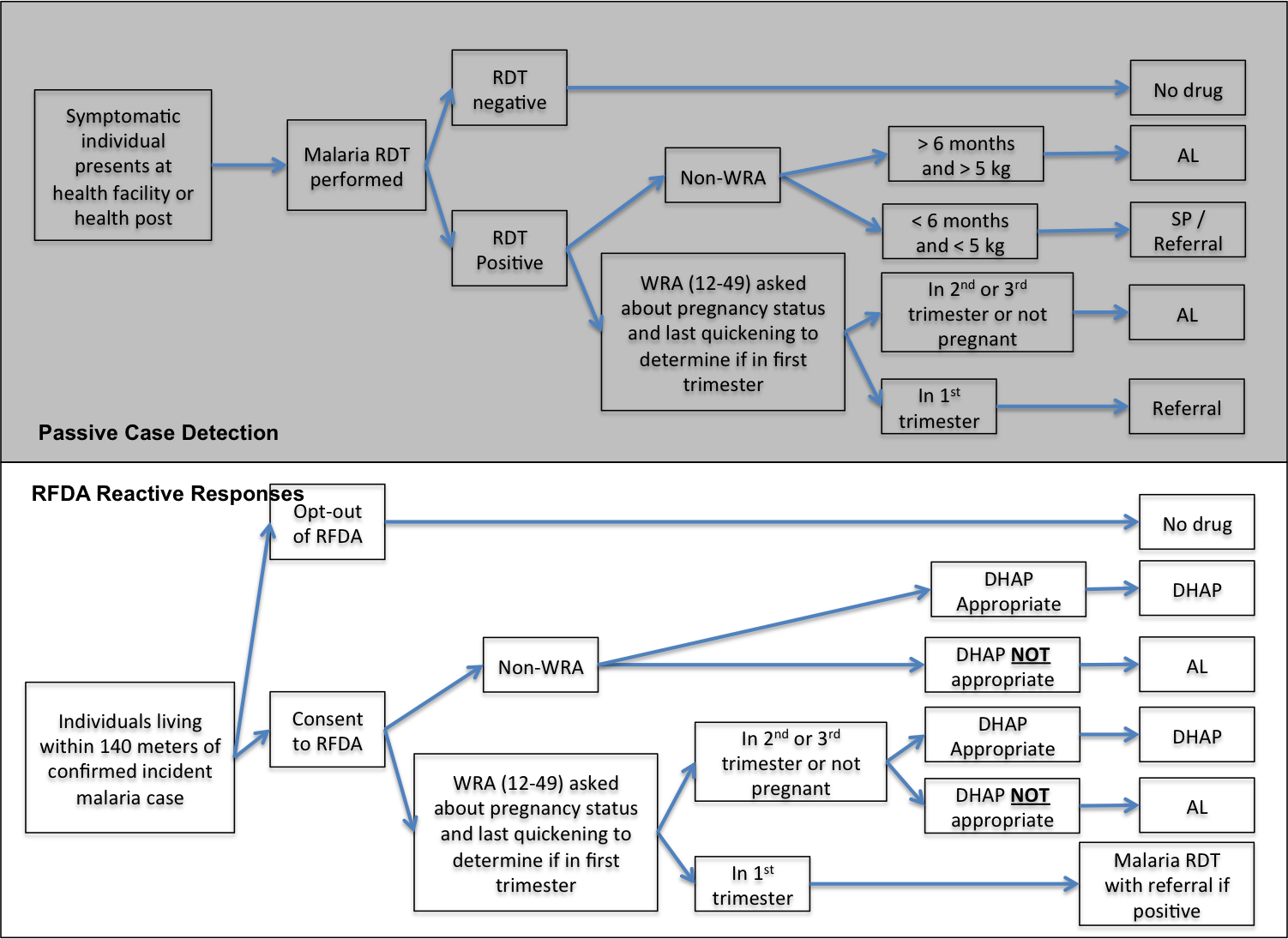


Figure 4 - Decision tree for patient interactions during passive case detection at the health center / health post and RFTAT with AL in the community. mRDT, malaria RDT; WRA, Women of Reproductive Age; HC, Health Center; AL, Artemether-lumefantrine; SP, Sulphadoxine-pyrimethamine.

### Dihydroartemisinin - piperaquine (DHAP)

DHAP has been approved by the Zambian Ministry of Health as the second-line standard of care drug for the treatment of uncomplicated malaria in Zambia (AL is currently the first-line treatment). Overall, DHAP is safe, effective and well-tolerated by individuals


^30,31^. A recent study of intermittent preventive treatment of children in Uganda found that monthly administration of DHAP to children in primary school was both safe and effective


^32^, and previously in Thailand monthly administration of DHAP to adults was found to be both safe and effective


^33^. However in Zambia the administration of DHAP will be limited to twice yearly with at least two months in between doses, in accordance with the manufacturer’s instructions.

A fixed dose of DHAP currently consists of combination tablets containing 40 mg of dihydroartemisinin and 320 mg of piperaquine. A target dose of 4 mg/kg/day dihydroartemisinin and 18 mg/kg/day piperaquine once a day for 3 days will be given.

#### Treatment notes

For this study, DHAP will be given in Eurartesim© tablets. While full product details are available from the manufacturers Sigma Tau (www.sigmatau.com), the following key issues are highlighted when administering DHAP, and summarised as a decision tree in Figure 5. DHAP is not recommended during the first trimester of pregnancy. Women of reproductive age (12-49) will be asked about their pregnancy status and if pregnant, the last time they felt the baby move. Any woman unsure of her pregnancy status will be offered a rapid pregnancy test, or referred to the nearest health facility. Women reporting that they are pregnant and have not felt the baby move (still in the first trimester), testing pregnant through the rapid pregnancy test or who are unsure of their pregnancy status will be tested with a malaria RDT and referred to the health center if testing positive. Pregnant women before the first trimester will not be treated with DHAP; pregnant women beyond the first trimester will be treated with DHAP. Additionally, DHAP should be administered with water no less than 3 hours after the last food intake, and no food should be taken within 3 hours after each dose. Any individual who does not wish to take, or cannot take DHAP, for whatever reason, will be offered the standard of care i.e. AL.

If a patient vomits within 30 minutes of taking DHAP, the whole dose will be re-administered. If a patient vomits within 30-60 minutes, half the dose will be re-administered. Re-dosing will not be attempted more than once. If the second dose is vomited, the patient will be referred to the nearest health centre. No more than two courses of DHAP will be given within a 12 month period, with a 2 month window between the two courses due to the long elimination half-life of piperaquine. Individuals identified during RFTAT responses who have been treated with DHAP in the previous 2 months, or twice within the previous year will be given AL.
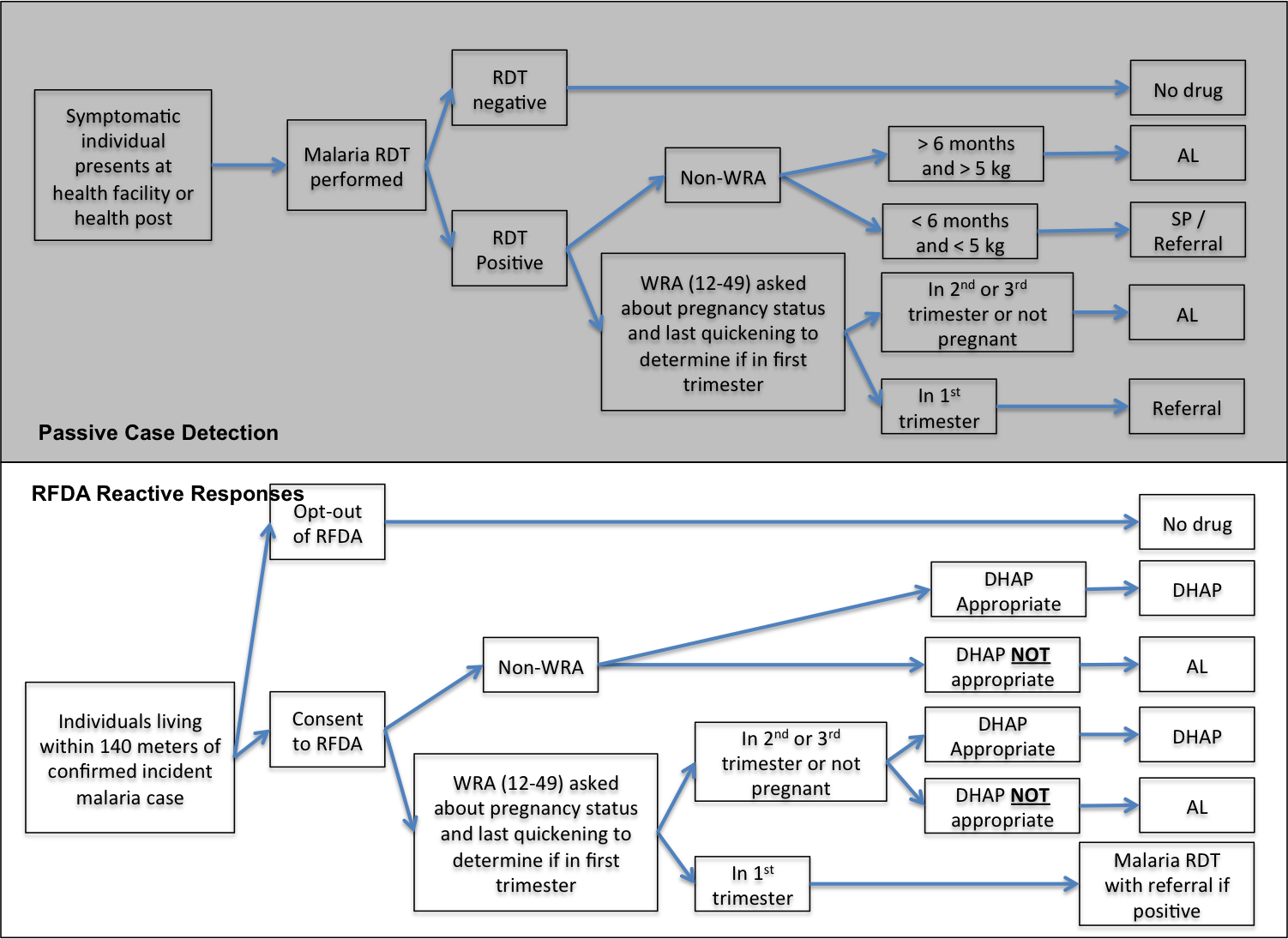


Figure 5 - Decision tree for patient interactions during passive case detection at the health center/ health post and RFDA with DHAP in the community. mRDT, Malaria RDT; WRA, Women of Reproductive Age; HC, Health Center; DHAP, dihydroartemisinin-piperaquine. *due to previous dosing schedule.

## Study participants

Study participants will consist of three groups selected from residents of the health center catchment areas randomized to the control or intervention arms and will be selected as follows:

1. Individuals in households selected for endline sampling. Households will be identified as describe in section 2.5 (page 23)
2. Individuals living within 140 meters of an index case selected for a CHW-led reactive response (Figure 6)
3. Individuals living within 140 meters of an index case selected for a research team supported reactive response (see Section 2.4.1 and Figure 7), who will receive additional follow-up visits 3, 30 and 90 days after the initial reactive response.

## Reactive responses

All reactive responses will aim to take place within 7 days following presentation of an RDT positive incident malaria case to a health center or health post within the study areas. Based on work showing the spatial distribution of malaria infections in Southern Province (D. Larsen dissertation, unpublished), all individuals residing within 140 meters of the index case household will be included in the reactive response. In the control (RFTAT) arm, the current standard of care will be provided in which individuals will be tested for malaria using an RDT. Positive individuals will be offered treatment with AL or referred to a health center according to national treatment guidelines. In the intervention (RFDA) arm, all individuals residing within 140 meters of the index case household will be offered DHAP. Testing with an RDT will not be performed in the RFDA arm, with the exception of research team responses (see Section 2.4.1 on page 20).


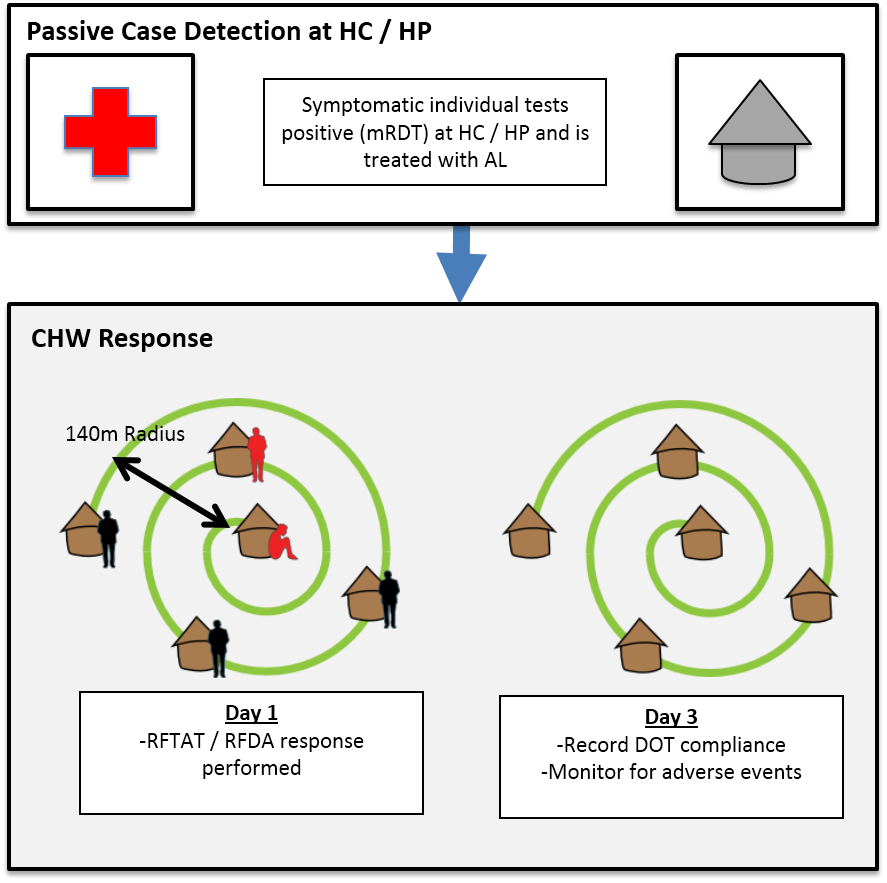


Figure 6 - Schematic of activities before and during a CHW reactive response. HC, Health Center; HP, Health Post; AL, artemether-lumefantrine. Seated / standing figures indicate individuals who are symptomatic or healthy respectively. The colour of individuals represents whether they are infected with P. falciparum (Red) or uninfected (black).

### Research Response

In order to assess parasite clearance and the risk of re-infection following a reactive response, a subset of index cases will be randomly selected for follow-up by a research team within each participating health center catchment area (Figure 7). Equal numbers of index cases will be randomly selected in each arm and in each clinic, with a minimum of two index cases per month in each arm i.e. minimum of four total per month. Only index cases reporting no history of travel will be enrolled. A questionnaire will be administered to all individuals participating in the reactive research responses and a finger prick blood sample will be obtained to collect dried blood spots (DBS) for study research purposes only. Participants will be adequately protected with risks minimized as much as possible during sample collection (see Section 3.1 for more information). Information collected through the questionnaire will include demographic information, current symptoms, recent malaria history, travel history, RDT result, HS RDT result (in RFDA arm only) and treatment administered (Appendix 6 and Appendix 7). Geocoordinates will be collected for each household targeted for a reactive response. Blood samples will be used to detect asexual stage parasites by PCR and all positive samples will be genotyped using a molecular bar code.

Follow-up visits will be conducted 3 (Appendix 9), 30 (Appendix 10) and 90 (Appendix 10) days after the initial reactive response. Additional information will be collected from each targeted individual on signs and symptoms of malaria, other treatments received, adherence to the drugs given (if any), and adverse reactions. A finger prick blood sample will be collected from each individual as a DBS at the 30 and 90 day follow-up visits. From the initial reactive response visit, blood samples will be used to detect asexual stage parasites by PCR and all positives genotyped using a molecular bar code.

For research responses enrolled in the RFDA arm, consenting individuals will be tested using the HS RDT as well as the standard RDT. No treatment decisions will be made based on the results from either the HS RDT or the standard RDT, rather they will all be offered DHAP. Individuals who do not consent to be enrolled in the RFDA arm will continue to receive the standard of care i.e. testing with a standard RDT and if positive, treatment given according to national policy.


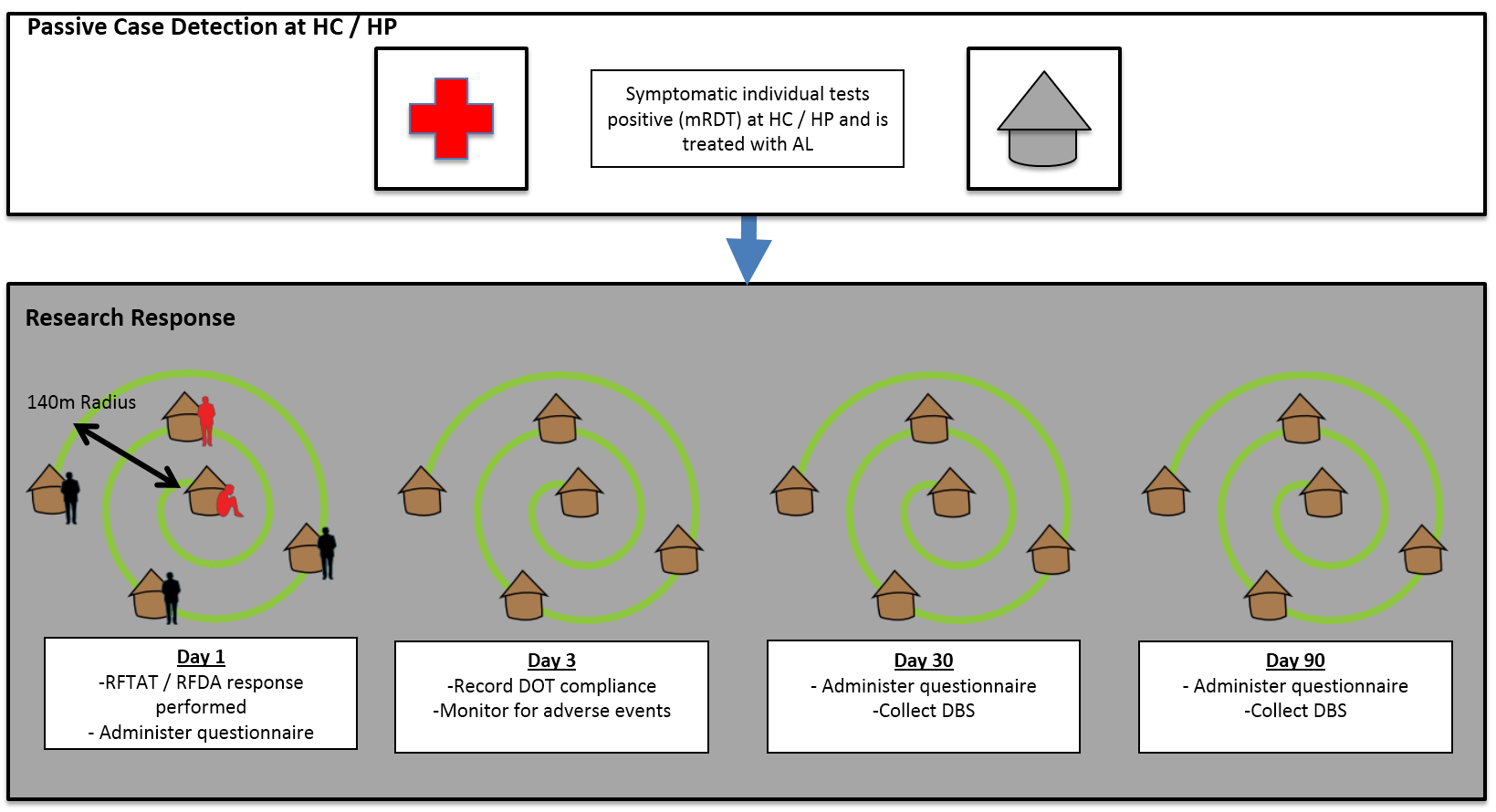


Figure 7 – Schematic of activities before and during a reactive research response. HC, Health Center; HP, Health Post; AL, artemether-lumefantrine; DBS, Dried blood spot; DOT, Directly observed treatment. Seated / standing figures indicate individuals who are symptomatic or asymptomatic, respectively. The colour of individuals represents whether they are infected with *P. falciparum* (Red) or uninfected (Black).

### Directly Observed Treatment (DOT)

DOT describes a system whereby patients taking a treatment dose are directly observed throughout the treatment course, thereby ensuring maximal compliance with the drug regime. In the context of malaria elimination, compliance is important for preventing the development of drug resistance and also fully removing the infectious reservoir from the population. To fully exploit the window of chemoprophylaxis, i.e. the period where drug levels protect treated individuals from being infected by an infectious mosquito bite, the entire target population needs to comply with the treatment regime. Therefore, a modified DOT schedule will be implemented wherein treatment is directly observed on day 1 and day 3, but not on day 2 for all individuals within a reactive response in the intervention and control arms. Treatment blister packs will be observed on Day 3 as a proxy of compliance for Day 2.

As stated in Section 2.2.2.1 on p17, DHAP needs to be taken on an empty stomach, and in certain cases this may prevent DOT being implemented.

## Surveys

The health centre catchment areas selected for this study will be enumerated fully prior to sampling for the end-line cross-sectional survey. Once randomization of health centers to each arm is complete, sampling buffers of at least 3km will be derived for each catchment in order to maintain an adequate distance between sampled households in differing study arms. The use of buffers for sampling is to prevent confounding of trial outcomes due largely to community level transmission effects crossing between study treatment groups. Such contamination could arise either due to the localized effects of the intervention on transmission geographically or due to mosquito movement between study areas. A second additional benefit of utilizing these sampling buffers will be to potentially reduce human cross-over between study areas. The sampling buffers will be derived based on geographic information system (GIS) layers mapping the health center catchment areas corresponding to areas administratively covered by a specific health center. These buffers will ensure that no households will be sampled within 3km of a study area which was randomized to a differing study arm. No sampling buffer will be applied where a catchment has been randomly allocated adjacent to another within the same treatment group, as in these situations there will be no difference in the intervention across the catchment boundary and no risk of cross-over/contamination between treatment groups.

Following complete enumeration of the study area, a simple random sample of households will be drawn from each catchment area as outlined in section 2.9 on p31, after restricting the sampling frame for sampling buffers in GIS. This process will be repeated for each survey time point.

A modified malaria indicator survey (Appendix 4) will be administered at the endline. The modified malaria indicator survey will include collection of the following information:

- Travel history
- Net ownership and use
- IRS history
- History of fever
- Treatment seeking behavior
- Household structure characteristics
- Household education
- Asset-based index to generate measures of wealth

The above information will be enable us to control for any differences between the selected health centre catchment areas.

All consenting individuals ≥ 1 month of age and < 15 years old in households participating in the surveys will be tested using an RDT and if positive given AL according to national treatment guidelines, or referred as appropriate e.g. if pregnant or suspected to be pregnant. Individuals will also be consented to provide finger prick blood samples (approximately 200 μL) collected as dried blood spots for:

- detection of asexual stage parasites by PCR
- serological responses to *P. falciparum* antigens
- serological responses to anopheline salivary peptides
- genotyping PCR positive samples using a molecular bar code

## Routine Data Reporting

Standard reporting of malaria incidence through passive cases presenting to community health workers and health clinics will be monitored and available for the study area and time period. This data is collected, aggregated - either weekly for Health Clinics or monthly for Health Posts, and then submitted by mobile phone. A complete list of data elements contained within the standard reporting is listed in Appendix 12.

## Consent

Written informed consent will be sought for each interaction. A summary of consent procedures is shown in Table 2. In the event that an individual refuses DHAP, the individual will be offered the standard of care in Zambia, which currently consists of RFTAT with an RDT and AL. To monitor refusal rates, the number of people who opt-out will be recorded.

Table 2 – Consent schedule and timing of data collection.

|  | Written informed consent for intervention | Written informed consent for DBS (finger stick) | Written informed consent for data collection |
| --- | --- | --- | --- |
| Endline Survey |  | X  (Appendix 3) | X  (Appendix 3) |
| Passive Case Detection |  |  |  |
| RFTAT intervention* |  |  |  |
| RFDA intervention | X  (Appendix 11) |  |  |
| Reactive Research Response (RFTAT / RFDA) | X  (Appendix 11 – RFDA Only) | X  (Appendix 8⁺) | X  (Appendix 8⁺) |
| - 3 day follow-up |  |  | X  (Appendix 8⁺) |
| - 30 day follow-up |  | X  (Appendix 8⁺) | X  (Appendix 8⁺) |
| - 90 day follow-up |  | X  (Appendix 8⁺) | X  (Appendix 8⁺) |

*Current routine strategy

⁺Individuals will be consented at the first visit for all subsequent interactions.

### Subject capacity

For individuals who do not have the capacity to provide consent for themselves, consent will be sought from the head of household or parent/guardian. If either is absent, the subject without capacity to provide individual consent will be excluded. If individuals are unable to sign consent forms, they may make a mark and a witness will attest to the subject’s consent.

## Research Strategy by Aim

### Primary Aim

Compare the effectiveness of RFDA using DHAP with RFTAT using AL in achieving zero seropositivity in children under 15 years of age.

Hypothesis: RFDA with DHAP will be more effective in reducing malaria prevalence in the community than RFTAT using AL by clearing parasites from RDT-negative individuals with subpatent parasitemia and through the longer chemoprophylactic protection conferred by DHAP.

**Design**: Cluster randomized controlled trial in which eight health center catchment areas are randomized to RFTAT with AL (control arm) and eight health center catchment areas are randomized to RFDA with DHAP (intervention arm)

**Population**: Residents (under fifteen years of age) of a random selection of households in health center catchments within the intervention and control arms using satellite imagery to define the sampling frame

**Outcomes**: 1) malaria seropositivity measured at endline;

**Inclusion/Exclusion criteria:** All consenting individuals aged ≥ 1 month and < 15 years will be tested for parasite prevalence in the survey

**Procedures**: 1) randomization; 2) RFTAT with AL or RFDA with DHAP; 3) endline survey

**Assays**: Serological analysis of DBS collected during endline survey

**Analysis**: For the outcome of seropositivity, a comparison of seropositivity will be made between intervention and control areas and between children stratified into younger (aged ≥ 1 month to < 2 years), middle (aged ≥ 2 years to < 5 years), and older (aged ≥ 5 years and < 15 years). To account for correlated data at the cluster level, mixed effects regression models with the health center as a random intercept will be the primary analytic method to estimate the effectiveness of RFDA compared to RFTAT for the outcome of malaria prevalence as measured through seropositivity. Analyses will account for environmental factors, socio-economic status, housing construction, education level, and demographic measures of the population as well ITN coverage and indoor residual spraying.

### Secondary Aim 1

Compare the effectiveness of RFDA using DHAP with RFTAT using AL in reducing RDT confirmed malaria incidence through passive case detection at health facilities

Hypothesis: RFDA with DHAP will be more effective in reducing malaria incidence in the community than RFTAT using AL by clearing parasites from RDT-negative individuals with subpatent parasitemia and through the longer chemoprophylactic protection conferred by DHAP.

**Design**: Cluster randomized controlled trial in which eight health center catchment areas are randomized to RFTAT with AL (control arm) and eight health center catchment areas are randomized to RFDA with DHAP (intervention arm)

**Population**: all residents of the health center catchment areas within the intervention and control arms presenting to a health facility with confirmed malaria by RDT or microscopy

**Outcomes**: 1) incidence of malaria confirmed by RDT or microscopy as measured through passive case detection at health posts and health centers

**Inclusion/Exclusion criteria:** Not applicable. Study will use aggregate health facility records of 16 health centers in the study.

**Procedures**: 1) RFTAT with AL or RFDA with DHAP; 2) routine passive case detection

**Assays**: None

**Analysis**: For the outcome of malaria incidence a Poisson or negative binomial regression with health center month as the unit of analysis will be generated. Total outpatient attendance will be used as an offset to account for treatment seeking behavior. Lagged environmental factors will be incorporated to account for seasonality of malaria transmission. Month of the year may be included to account for temporal autocorrelation.

### Secondary Aim 2

Compare the effectiveness of RFDA using DHAP with RFTAT using AL in reducing the prevalence of malaria and preventing re-infection in individuals receiving reactive responses.

Hypothesis: Among treated individuals, parasite clearance will be similar between those receiving RFTAT using AL and those receiving RFDA with DHAP. However, overall parasite prevalence following a reactive response will be lower in areas receiving RFDA with DHAP than in areas receiving RFTAT with AL because of clearance of subpatent infections and the longer protective effect of DHAP.

**Design**: longitudinal cohort study

**Population**: residents within the intervention and control arms receiving a reactive research response

**Outcomes**: 1) PCR parasite prevalence at 0, 30 and 90 days following a reactive research response

**Inclusion/Exclusion criteria:** All consenting individuals aged ≥ 1 month will be tested for parasite prevalence 0, 30 and 90 days following the reactive research response. Individuals to be included in the assessment of secondary aim 2 will have parasite measurements at 0, 30 and 90 days following the reactive research response.

**Procedures**: 1) reactive response; 2) follow-up visit at 30 days; 3) follow-up visit at 90 days

**Assays**: PCR for asexual parasites using DBS collected at 0, 30 and 90 days

**Analysis**: Parasite prevalence at 30 days and 90 days will be analyzed separately. Areas receiving a reaction will form the unit of analysis while malaria infections during returns to the areas receiving a reaction will be measured as a count variable. A mixed effects Poisson regression with the offset being the number of individuals providing filter paper with a DBS during the return to the areas receiving a reaction will be used to compare the effectiveness of RFDA v. RFTAT at clearing an area of malaria infections (30 days) and preventing reintroduction following the reactive response (90 days). A number of factors will be tested for acting as effect modifiers through a test for interaction including initial parasite prevalence at day 0 during the response, response coverage in the area, asset-based wealth index, vector control coverage of the area, age and demographics of the individuals living in the area, as well as environmental factors such as vegetation cover and topographical measures. A fixed effects regression approach, wherein the difference between the number of malaria-infected individuals at day 0 during the reaction and the number of malaria-infected individuals at day 30 and/or day 90 following the reaction may be used to assess how the malaria parasite reservoir changes following the reactive responses.

### Secondary Aim 3

Compare the cost-effectiveness of RFDA using DHAP with RFTAT using AL in reducing the prevalence of malaria in the community.

Hypothesis: RFDA will be more cost-effective in reducing malaria prevalence than RFTAT by clearing parasites from individuals with subpatent parasitemia, through the longer protective effect conferred by DHAP, and the cost savings of not using a diagnostic test (RDT).

**Design**: cluster randomized trial

**Population**: all residents of the health center catchment areas within the intervention and control arms

**Outcomes**: Costs associated with RFTAT and RFDA, including personnel, transportation, and commodities.

**Inclusion/Exclusion criteria:** Not applicable. Study will use cost estimates and estimates of reduction in parasite prevalence as measured in Primary Aim.

**Procedures**: 1) randomization; 2) RFTAT with AL or RFDA with DHAP; 3) follow-up visit on day 3 to assess adverse reactions; 4) measurement of costs

**Analysis**: Detailed tracking of program expenditures on the establishment, production and delivery components of the RFDA and RFTAT interventions, including the value of time spent by the CHWs and other staff, will be used to calculate the financial costs. Costs will be calculated using an ingredients approach that involves enumerating both the quantity of specific inputs (e.g., hours spent, number of rapid diagnostic tests used, etc.) and the time spent during the intervention. As the analysis is intended to be incremental, existing infrastructure and recurrent inputs that would be present in the absence of the intervention will not be costed. The emphasis of the cost analysis is on determining the cost of RFDA and RFTAT alone, and not assessing the cost of training CHWs in the diagnosis and treatment of malaria nor in the follow-up visits to areas receiving reactive responses. Cost-effectiveness will then be measured through incident malaria cases averted as measured through the difference in malaria incidence at health centers in RFDA and RFTAT arms.

### Secondary Aim 4

Measure the proportion of *P. falciparum* infections likely attributable to importation and local transmission using parasite genotyping as well as defining genotypes spatial distribution.

Hypothesis: The proportion of *P. falciparum* infections attributable to importation will increase in both the intervention and control arms as local transmission is interrupted through the reactive case detection strategies. Additionally, the genetic diversity of locally transmitted parasites will decrease as transmission decreases but will be lower in communities receiving RFDA.

**Design**: nested cross-sectional study

**Population**: All individuals with a positive RDT for *P. falciparum* identified in the endline survey and during reactive case detection and their evaluation in reactive research responses.

**Outcomes**: Parasite population genetics, and spatial distribution of defined parasite genotypes.

**Inclusion/Exclusion criteria:** All individuals tested for malaria during the endline survey as well as during reactive research responses who have consented to further analyses of their dried blood spot sample.

**Procedures**: Sample collection from 1) reactive responses; 2) endline survey; genotyping of PCR-positive samples

**Assays**: Genotyping of *P. falciparum* genomes using PCR-positive DBS

**Analysis**: All malaria infections will be geo-referenced through data collection. Spatial variation in the genomic complexity and diversity will be estimated using the Geneland


^34^package in R


^35^ which uses Bayesian methodology to identify clusters of individuals with similar genotypes while accounting for spatial dependence of geo-referenced data. The procedure is capable of analyzing multivariate gene outcomes including SNPs. The number of separate genetic populations in the study area will be estimated and mapped; migrants (*P. falciparum* infections dissimilar genetically from others in nearby space) will be classified as imported malaria cases without onward transmission and will also be identified and mapped. The influence of a number of environmental factors on the spatial distribution of malaria parasites will be estimated. The enhanced vegetation index available through remotely sensed data will provide an indicator of adult mosquito habitat


^36,37^. A digital elevation model will be used provide an indicator for the propensity of an area to harbor mosquito breeding sites


^38–40^. We aim to utilise spatio-genetic methods that have been utilized in various aspects of ecology, but have not yet been applied to malaria epidemiology to understand transmission dynamics in the two arms


^41^.

Genetic complexity, defined as the number of genetically unique parasites found within health centre and health post catchments, will be compared between RFDA and RFTAT areas using a Poisson or linear regression depending upon the number of genetically unique parasites found. A number of factors will be tested including proximity of the area to the main road, wealth of the area, vector control coverage of the area, age and demographics of the individuals living in the area, as well as environmental factors such as vegetation cover and topographical measures.

### Secondary Aim 5

Assess the utility of serology to measure short-term changes in malaria transmission and evaluate malaria elimination programs

Hypothesis: Different serological outcomes will be both time sensitive and more prevalent than detected malaria parasite infections leading to less costly evaluation of malaria elimination programs

**Design**: nested longitudinal study

**Population**: all residents enrolled for a reactive research response within the intervention and control arms

**Outcome measures**: Antibody responses to appropriate *P. falciparum* antigens, e.g. AMA1 and MSP_1-19_.

**Inclusion/Exclusion criteria:** All individuals tested for malaria during the endline survey as well as during reactive research responses who have consented to further analyses of their dried blood spot sample.

**Procedures**: 1) randomization; 2) endline survey; 3) measurement of antibodies to *P. falciparum* antigens

**Assays**: Enzyme immunoassay to measure IgG antibodies to *P. falciparum* antigens from DBS

**Analysis**: The correlation between malaria parasite prevalence and various estimates of serology will be assessed in the endline survey samples. The analyses listed in the primary aim assessing the effectiveness of the intervention to reduce malaria parasite prevalence and malaria incidence will be repeated with the outcomes of serology and effect estimates will be compared.

The spatial variation of malaria parasite prevalence will be compared with the spatial variation of individuals measuring exposure to *Anopheles* spp. antigens (see below).

### Secondary Aim 6

Assess the feasibility of using remotely sensed malaria risk maps to identify areas with higher potential for local malaria transmission

Hypothesis: Exposure to anopheline mosquito salivary peptides as measured by serology will be correlated with the spatial distribution of malaria parasite infections and can be predicted through mapping mosquito habitat

**Design**: nested case-control study

**Population**: all residents of reactive research responses within the intervention and control arms. Cases will be defined as individuals testing positive for malaria during a reactive research response. Controls will be defined as individuals testing negative for malaria during a reactive research response.

**Inclusion/Exclusion criteria:** All individuals tested for malaria during reactive research responses.

**Outcome measures**: Antibodies to anopheline salivary peptides

**Procedures**: 1) randomization; 2) endline survey; 3) measurement of antibodies to anopheline salivary peptides

**Assays**: Enzyme immunoassay to measure IgG antibodies to anopheline salivary peptides using DBS

**Analysis**: The association between the spatial location of a household of an individual having a malaria infection and the spatial location of individuals being exposed to anopheles mosquitoes as measured through serological reaction to anopheline salivary peptides will be measured using an odds ratio. Three risk surfaces will be generated using kernel intensity functions. First, a simple risk surface of malaria infections compared to non-infected individuals will be generated using an adaptive bandwidth


^42^. Second, a simple risk surface of exposure to anopheline salivary peptides compared to unexposed individuals will be generated using an adaptive bandwidth


^42^. Third, a more complex risk surface will be modeled from remotely sensed *Anopheles* breeding sites based on topographical indices derived from remotely sensed digital elevation models


^38–40^. This more complex risk surface will utilize a kernel intensity function with a fixed bandwidth representing mosquito dispersal based on ongoing literature review of mark-release-recapture studies. The similarity between risk surfaces will be measured through estimating the correlation of pixel values at the location of each individual. We will compare the geographic footprint of clusters of malaria infection and proximity to remotely sensed *Anopheles* breeding sites.

### Secondary Aim 7

Assess the utility of a HS RDT in identifying additional standard RDT sub-patent infections in the RFDA arm

Hypothesis: Reducing the limit of detection will enable additional sub-patent infections to be identified

**Design**: nested cross-sectional study

**Population**: Residents within the control (RFDA) arm receiving a reactive research response

**Inclusion/Exclusion criteria:** All consenting individuals enrolled during reactive research responses in the RFDA arm

**Outcome measures**: Incidence of malaria from standard RDT, HS RDT, *Pf* HRP2 Quansys Q-plex ELISA (Logan, Utah; Q-plex ELISA) and PCR

**Procedures**: 1) reactive response in RFDA arm

**Assays**: HS RDT and standard RDT on consenting individuals in the field, PCR (including *Pf* HRP2/3 gene deletions) and Q-plex ELISA on DBS

**Analysis**: The HS RDT performance will be measured by sensitivity, specificity, negative and positive predictive values (NPV, PPV) against RDT, PCR, and Q-plex ELISA results. The data from all assays / tests will be used in descriptive analyses including the distribution of parasitemia (PCR), HRP2 (Q-plex ELISA), *Pf* HRP2 detection by RDT and HS RDT, and if present, the prevalence of *Pf* HRP2/3 deletions.

Although abundant during infection, the half-life of HRP2 is long and can remain present despite parasite clearance by treatment; as a result, there is a need to define false positives by PCR and also by an independent *Pf* HRP2-based assay. The Q-plex ELISA contains spots for *Pf* HRP2 and Pan-malaria LDH. The capture and detection antibodies are orthologous to those on the RDTs and therefore confirm the presence or absence of *Pf* antigenemia. The Q-plex ELISA also quantifies both *Pf* HRP2 and Pan-LDH. The *Pf* HRP2 measurement, as well as the standard PCR detection results, will be used to further define the limit of detection of *Pf* HRP2 and parasitemia respectively by the HS RDT in an endemic area. Additionally, for DBS specimens that are positive for *Pf* by qPCR, but negative by all *Pf* HRP2-based tests/assays (RDT, HS RDT, and Q-plex ELISA), a *Pf* HRP2 and *Pf* HRP3 (HRP2/3) deletion PCR will be performed.

### Secondary Aim 8

We will determine the direct and spillover effects of the RFTAT / RFDA interventions and assess whether effects vary by intervention coverage, distance to intervention, and time from incident case detection. We will then pool the data with two other similar studies from Namibia and Swaziland and perform a meta –analysis of the outcomes.

Hypothesis: Reactive case detection impact is not limited to the radius of the intervention, but is anticipated to be weak therefore data needs to be pooled to increase the power and maximize the likelihood of identifying spillover effects.

**Design**: longitudinal cohort study, primary health facility incidence data

**Population**: all residents within the study area

**Outcomes**: 1) PCR parasite prevalence at 0, 30 and 90 days following a reactive research response, 2) health facility incidence data

**Inclusion/Exclusion criteria:** All consenting individuals aged ≥ 1 month will be tested for parasite prevalence 0, 30 and 90 days following the reactive research response.

**Procedures**: 1) reactive response; 2) follow-up visit at 30 days; 3) follow-up visit at 90 days 4) health facility reported incidence data

**Assays**: PCR for asexual parasites using DBS collected at 0, 30 and 90 days

**Analysis**: We will estimate direct effects and spillover effects of reactive, focal malaria elimination interventions on Plasmodium falciparum malaria incidence and prevalence and assess whether effects vary by intervention coverage, distance to intervention, and time from incident case detection. The direct effects analysis will include individuals/ households that received interventions, and the spillover effects analysis will include those who did not receive interventions but are in the study area. Parameters will compare outcomes in each group between arms overall and conditioning on intervention coverage, distance to intervention, and time from incident case detection. We will perform an individual participant meta-analysis. First we will estimate effects in each of three cluster-randomized trials; then we will pool effects using fixed and random effects.

## Survey Sample Size

The endline survey samples will provide malaria seropositivity parasite prevalence information for approximately 500 children per RHC catchment area (150 ≥ 1 month and < 2 years; 150 ≥ 2 years and < 5 years; 200 ≥ 5 years and < 15 years). Seropositivity in control arms is assumed to be at least equal to a parasite prevalence (as measured by RDT) of 5%. An inter-cluster variation of 0.5


^43^, was found in the mass screening and treatment campaign in the districts adjacent Lake Kariba and was used for this sample size calculation. Including 8 health centers per intervention arm would allow for detection of a 64.5% relative difference between intervention arms with 80% power and 95% specificity (decrease from 5% seropositivity to 1.78 %).

Table 3 shows a sensitivity analysis of the detectable relative difference in intervention arms when varying the assumptions for seropositivity and inter-cluster variation with the midpoint estimate in bold.

Table 3 - Sensitivity analysis of detectable relative difference in seropositivity between intervention arms when varying seropositivity and the inter cluster variation while assuming 8 health centers per intervention arm and 500 children sampled per health center catchment. Figure in bold represents best estimates of parameters.

| Inter cluster variation | Seropositivity | | | | |
| --- | --- | --- | --- | --- | --- |
|  | 1% | 2.5% | **5%** | 7.5% | 10% |
| 0.3 | 64.5% | 50.7% | 44.6% | 42.3% | 41.1% |
| 0.4 | 69.7% | 58.0% | 53.1% | 51.4% | 50.4% |
| **0.5** | 75.7% | 65.7% | **61.7%** | 60.3% | 59.6% |
| 0.6 | 82.3% | 73.7% | 70.4% | 69.2% | 68.6% |
| 0.7 | 89.5% | 81.9% | 79.1% | 78.1% | 77.6% |

## Study Logistics

### Community Mobilization and Sensitization Activities

Community mobilization and sensitization activities will be conducted prior to the implementation of activities. These activities will follow similar methods as deployed prior to Zambia’s MIS and would prepare communities for the reactive responses, including collection of a finger stick blood sample for parasite and antibody testing. A general information letter and accompanying flyer will be prepared for participating districts and local communities. These documents will include information about the purpose, procedures, and importance of household participation. Further, a radio spot will be developed and aired on local community radio stations with service areas matching the selected communities. The radio spot will serve to introduce the activity and encourage participation. PATH already has local knowledge and key relationships with leaders in the study areas. Thus, existing communication channels with key leaders e.g. chiefs and headmen, will be leveraged to share information about the benefit of the upcoming research activities.

### Incentives

Incentives for CHWs (standard Step D):

- Bicycles
- Basic feature phones available as an incentive / trade-in for completion of Step D monthly reports
- Talk time sent automatically as an incentive for completion of Step D monthly reports. Value will reflect the timeliness of the reporting
- CHW packs - backpack with sharps disposal container

## Data Management

Data collected during the three surveys and the reactive research responses will be recorded on an android smartphone or tablet. This personally identifiable data will be securely transferred over the telephone network, as soon as a data connection is available, to a central server controlled by PATH. Access to the server will be password protected and secured according to best practice. Local copies of the database will be secured in password protected files on password protected computers.

Prior to analysis, data will be de-identified with the exception of geo-location codes which are necessary for specific per-protocol analyses, the absence of individual identifying information will protect subject confidentiality.

All paper records, consent forms and biological specimens will be stored in a locked location.

## Personnel

### Survey team

Teams consisting of the individuals listed below will be recruited to perform the endline survey. Training will occur in consultation with and oversight by National Malaria Control Program representatives.

- Local CHW to provide local knowledge of area and community residents
- Sample collector with previous experience collecting DBS
- Enumerator trained to enter data electronically
- Driver

### Standard CHW Response (RFTAT / RFDA)

CHWs will perform all required activities as per existing Step D documentation through the NMCP (Appendix 1).

### Research Response (RFTAT / RFDA)

Community health workers will inform the research team through an SMS text message upon detecting a confirmed incident malaria case. The research team will then determine whether to accompany the CHW on the reactive response based on the location of the incident case, ability to respond and number of responses previously performed in that area. Each response team will consist of three individuals:

- A sample collector for dried blood spots
- An enumerator to administer surveys and input data into the phone / tablet
- A supervisor to co-ordinate responses and support sample and data collection needs as required

Each research team will have access to a hired 4x4 vehicle as required as well as a project purchased motorbike.

It is anticipated that a total of four research teams will be required. Teams will be based in locations according to the geographical spread of the health centers. Each team will be able to respond to either arm (RFTAT / RFDA) to maximize flexibility in the workforce.

## Sample Analysis Assays

Samples collected during this trial will be stored in a plastic bag with silica gel before transport to Lusaka where they will be put in a freezer. All samples will be analysed at the National Malaria Control Centre, Zambia. A subset of samples i.e. those enrolled in the HS RDT analysis (maximum 3,000 samples) will be sent outside the country to the PATH laboratory (Seattle, Washington, USA) for additional analyses e.g. *Pf* HRP2 Quansys Q-plex ELISA testing (Logan, Utah; Q-plex ELISA) and *Pf* HRP2/3 deletion PCR, as these analyses cannot currently be performed in country. It is anticipated that DBS specimens will be shipped to PATH every 3-6 months once an MTA has been approved. Samples will be stored for a maximum of three years from the completion of the study.

### PCR analysis for presence of Plasmodium falciparum

DNA will be extracted from dried blood spots collected during this trial and tested for the presence of *P. falciparum* DNA using a sensitive nested PCR to the multi-copy locus, 18s rRNA, or other target as appropriate


^44,45^.

### Genotype analysis on positive samples

PCR positive *P. falciparum* samples will be genotyped by molecular barcode


^46^. Depending on the genetic diversity of the samples all or a subset of the 24 SNPs will be employed to generate a unique barcode for each parasite. If mixed infections consist of a high multiplicity of infection (MOI) i.e. they contain a large number of different parasite genomes, it may be necessary to increase the system resolution by moving to a 96-SNP based system.

### Serology

Serology on all samples collected during the endline survey will be conducted for estimates of both long (e.g. AMA-1) and short-term exposure antigens, recent parasite transmission (Csp), as well as cumulative exposure to *Anopheles* mosquitoes (salivary peptides).

## Challenges and Limitations

### False Positive Test Results

As incidence reduces, the positive predictive value of RDTs decreases (Figure 8). This means that as malaria prevalence approaches zero, the proportion of positive RDT results that will be false positive increases. We will measure the positive and negative predictive values of the RDT by comparing the results to detection of asexual stage parasites by PCR.


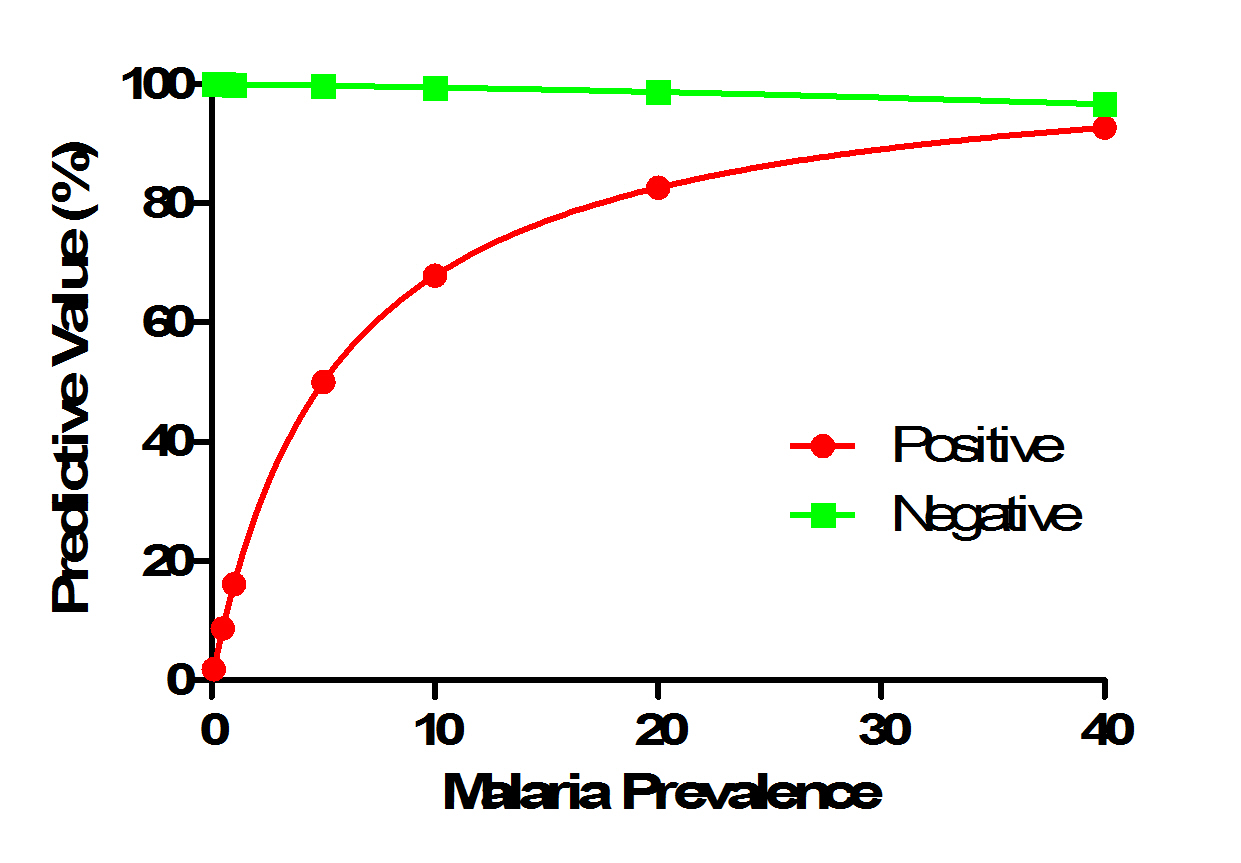


Figure 8 – Predictive value of a positive or negative RDT result across a range of malaria prevalence. Figures are calculated assuming a diagnostic test sensitivity and test specificity of 95% each.

### Adequate RDT and ACT Supplies

To ensure that there are no stock outs of RDTs or ACTs, commodity supply will be closely monitored through the routine reporting of health center (Step 1) data in DHIS2. A constant supply of commodities to ensure the ability to produce an unbiased estimate of the effectiveness of the interventions will be necessary. To that end, 3 months’ supply of RDTs and ACTs will be procured specifically for this project and will be retained to act as an emergency reserve for the health center catchment areas.

# Ethical Issues

Community surveillance using the Step D, test and treat strategy (control arm) has already been implemented throughout the majority of Southern Province and is the standard of care in Zambia. Focal drug administration for malaria control has not yet been implemented in Zambia although multiple examples exist around the world.

There may be physical risks to the participants/respondents and those receiving the intervention and the surveys associated with a finger stick from blood drop collection, but they are not considered more than minimal. Lancets or needle sticks are potentially, but only temporarily, painful. The risk to data collectors of needle sticks will be minimized by training, use of sterile lancets and proper disposal. This protocol will be reviewed by Western Institutional Review Board and the University of Zambia Institutional Review Board.

## Adequacy of protection against risks

We will administer an informed written consent and assent form in English or the local language as required by the participant. Table 4 outlines the exclusion criteria, inclusion ages and consent / assent forms that will be administered. For the household survey, written consent for participation in the survey and intervention will be provided by an individual who is at least 18 years of age according to the laws of Zambia. These forms will be read and explained or will be given to participants to read themselves and will include a full description of voluntary participation, the right to withdraw from the study at any time and the right to not answer any question. The forms will also address the risks, benefits and purpose of the intervention and study, including what we hope to learn. For individual’s ≥1 month and less than 18 years of age, consent will be sought from the head of household or parent/household guardian above age 18. For children greater than six years and less than 18, oral assent will be sought from the child. We will train all interviewers extensively on the consent and assent procedures. Checks in the field by the project team, including observation of a reactive response, assessment of completed consent statements and questioning of the interviewer will ensure the consent process is strictly followed. The confidentiality procedures (listed in section 2.11 on p33) are designed to meet all contingencies so that the privacy of the participants is preserved.

| **Study/intervention data collection** | **Exclusion** | **Inclusion Ages** | **Consent/Assent** |
| --- | --- | --- | --- |
| **Endline Survey** | < 1 month or >15 years of age | ≥1 month and <6 years old | Adult Consent (Appendix 3) |
|  |  | 6-<15 years old | Oral assent (Appendix 19) |
| **RFTAT intervention** | Severe malaria patients, First trimester pregnant women, < 1 month of age | ≥3 months old | DHAP Consent (Appendix 11) |
| **RFDA intervention** | Severe malaria patients, First trimester pregnant women, < 3 months of age, individuals contraindicated for DHAP | ≥3 months old  ≥1 month old | Oral Assent 6-17 years (Appendix 16) |
|  |  |  | Reactive research response (Appendix 8) |
| **Research around reactive responses for RFTAT and RFDA** | < 1 month of age | ≥1 month old | Oral Assent 6-17 years (Appendix 16 |
|  |  |  |  |

Table 4 - Summary of inclusion / exclusion criteria and consent/assent forms. * RFTAT with AL represents current standard of care so consent / assent will not be required.

The development of drug resistance has been linked to the administration of therapeutic doses of antimalarials


^11^. Our proposed strategy to reduce the risk of the development of drug resistance includes:

- Directly observed treatment of the first dose of DHAP during all reactive responses
- In research responses, the team will additionally:
  - Check blister packs on day 3 to ensure that the second dose was taken
  - Directly observed treatment of the third dose of DHAP on day 3

Our proposed strategy to reduce risks associated with finger sticks includes:

- To clearly explain the procedures to each individual or caretaker so that they understand the transient pain associated with collection of finger prick blood samples and the extremely low risk of infection.
- To use specially trained and supervised fieldworkers who will be able to answer commonly asked questions.
- To specially train fieldworkers to collect and handle biological specimens and ensure that all data entry personnel are trained on confidentiality and safety issues, as well as on informed consent procedures. This includes special training for all team members on universal precautions for handling biological specimens.

DHAP has been approved by the Zambian Ministry of Health as the new alternate first-line standard of care drug for the treatment of uncomplicated malaria in Zambia. AL is also the first-line treatment for uncomplicated malaria and is widely available. A new Cochrane Review (2014) has shown that studies comparing DHAP to other antimalarials to be safe, effective and well-tolerated by individuals


^47^. DHAP is a combination of two antimalarial drugs (dihydroartemesinin and piperaquine). Dihydroartemesinin has been associated with the following: prolonged QT intervals, falciparum malaria, influenza, cough, fever, abnormal cardiac conduction, necrotizing enterocolitis in foetus or new-borns, leukocytosis, neutropenia, thrombocytopenia, hepatitis and seizure. The risk of DHAP is assumed similar to that of AL, the current widely available national first line treatment for uncomplicated malaria.

Adverse reactions to both RFTAT with AL and RFDA with DHAP will be assessed 3 days following the reactive responses when the CHW or research team checks for drug compliance. Percentage of administrations causing adverse reactions will be calculated as the number of adverse reactions detected divided by the number of administrations in the health center catchment. Risk factors for adverse reactions such as age and sex will be examined as needed. The key to managing side effects and potential adverse events (AEs) and serious AEs (SAEs) is through thorough training of CHWs, health facility staff and supervisors, and intensive sensitization of the community. CHWs will monitor their respective communities for AEs and refer any potential SAEs to designated health facilities. Health workers at these facilities will be informed of the program, potential side effects, and provided with AE reporting forms (Appendix 13). Cases that cannot be treated at health centers will be referred to nearest district referral centre or district hospitals, with transport provided by the study.

Any and all potential AEs following treatment with DHAP will be recorded through passive case detection at all local public and private health facilities in the target areas, including referral by CHWs. A data safety monitoring board (DSMB) will be established to oversee and report on any AE and SAEs that are potentially linked to the administration of DHAP, and as outlined in further detail in Section 3.2. The following steps will be taken for all SAEs that present to health facilities:

1. The health facility will complete an AE description form (Appendix 13). The completed form will then be submitted to the DSMB, study coordinator and principal investigator within 72 hours from the identification of the AE/SEA.
2. The DSMB will complete an AE investigation form (Appendix 14) to a) assess if the event is an SEA, and 2) to determine if the AE/SEA was caused by the administration of DHAP as part of this study within 24 hours.
3. The DSMB will provide the completed AE investigation form to the PI, who will submit to the Research Ethics Committees (RECs) at UNZA and PATH. The respective RECs will be notified within 24 hours of the DSMBs completion of the AE investigation form for events determined to be SEAs, and within 48 hours of DSMB notification if deemed an AE, but not an SEA.

Our proposed strategy to reduce risks associated with DHAP in pregnancy (first trimester) include:

- Before providing any woman of reproductive age (12-49) with DHAP we will first ask women if they are pregnant or potentially pregnant
- If a woman reports being pregnant, to determine the trimester of the pregnancy, in addition to asking how many months pregnant she may know she is, she will be asked about the date of her last menstruation and, if necessary, if quickening has occurred (i.e. if she has felt the baby move inside of her)
- To offer women that are unsure of their pregnancy status or who are potentially pregnant a rapid pregnancy test to confirm pregnancy or not
- To clearly explain to women the potential risk of taking DHAP during the first trimester of pregnancy and allow them to opt out of receiving DHAP while still receiving the standard of care in Zambia (the RFTAT intervention)

## Data and safety monitoring plan

An independent data safety monitoring board (DSMB) will be established consisting of at least 5 independent experts in malaria control, diagnosis, case management, biostatistics and epidemiology and one member of the research team who will receive monthly reports of adverse events during the intervention period of the trial. The charter will include trial stopping rules for safety. No data on futility or benefit will be determinable during the course of the trial as seasonality of malaria transmission timing of outcome data collection preclude developing stopping rules based on outcome data collected during the duration of the implementation of the intervention. Safety concerns associated with the wide scale use of DHAP, although unexpected, will form the basis of development of a stopping rule. The stopping rules for this trial will be based on detection of a significantly higher rate of mortality, hospitalization for possible drug related events, or any other severe adverse event in the RFDA intervention arm compared to expected rates in the control group.

In addition to the DSMB, a regional monitor will be used for supervision of study progress and adverse events. A study pharmacist from the provincial health office will also be used to manage and account for the supply of DHAP used in the study.

## Potential benefits of the proposed research to the participants and others

The selected areas as a whole and the children within the selected areas specifically will benefit from reductions in the burden of malaria disease as a result of the decreased reservoir of asymptomatic parasitemia. The development of novel strategies to further reduce malaria transmission beyond the capacity of insecticide treated nets and case management is essential to proceed toward malaria elimination.

RFDA provides additional benefit beyond the standard RFTAT intervention through the treatment of subpatent asymptomatic malaria infections and the provision of chemoprophylaxis in areas with recent malaria transmission.

## Collection of specimens

Each specimen upon collection will be given a unique id that contains no personally identifiable information. Participants data linked to this identification number will only be accessed by the principal investigators or with permission from the principal investigator. Data stored on paper will be stored in a locked room. Data stored electronically will be saved to a password-protected computer.

## Protocol Accessibility

The complete protocol will be registered at clinicaltrials.gov, ahead of intervention delivery in the study site. This will prevent the likelihood of undeclared post-hoc changes to the protocol following IRB submission and of selective outcome reporting when the trial results are published. Additionally, this will provide peers and relevant partners working in the arena of malaria control and elimination, advanced warning of a trial being planned for to prevent duplication of efforts where the outcomes of this trial may hold external validity for their given malaria transmission setting.

# Timeline

|  | **Year 1** | | | | | | | | | | | | **Year 2** | | | | | | | | | | | | **Year 3** | | | | | | |
| --- | --- | --- | --- | --- | --- | --- | --- | --- | --- | --- | --- | --- | --- | --- | --- | --- | --- | --- | --- | --- | --- | --- | --- | --- | --- | --- | --- | --- | --- | --- | --- |
| **Activity/Month** | **1** | **2** | **3** | **4** | **5** | **6** | **7** | **8** | **9** | **10** | **11** | **12** | **1** | **2** | **3** | **4** | **5** | **6** | **7** | **8** | **9** | **10** | **11** | **12** | **1** | **2** | **3** | **4** | **5** | **6** | **7** |
| Submission to IRB |  |  |  |  |  |  |  |  |  |  |  |  |  |  |  |  |  |  |  |  |  |  |  |  |  |  |  |  |  |  |  |
| Recruitment and training of program staff |  |  |  |  |  |  |  |  |  |  |  |  |  |  |  |  |  |  |  |  |  |  |  |  |  |  |  |  |  |  |  |
| Sensitization of communities / chiefs / district personnel |  |  |  |  |  |  |  |  |  |  |  |  |  |  |  |  |  |  |  |  |  |  |  |  |  |  |  |  |  |  |  |
| Recruitment and training of CHWs, Health center staff and district personnel |  |  |  |  |  |  |  |  |  |  |  |  |  |  |  |  |  |  |  |  |  |  |  |  |  |  |  |  |  |  |  |
| Recruitment and training of survey teams |  |  |  |  |  |  |  |  |  |  |  |  |  |  |  |  |  |  |  |  |  |  |  |  |  |  |  |  |  |  |  |
| Consent process of research areas |  |  |  |  |  |  |  |  |  |  |  |  |  |  |  |  |  |  |  |  |  |  |  |  |  |  |  |  |  |  |  |
| Collection of cohort data / samples in research areas |  |  |  |  |  |  |  |  |  |  |  |  |  |  |  |  |  |  |  |  |  |  |  |  |  |  |  |  |  |  |  |
| Lab analysis of samples (DNA / Ab) |  |  |  |  |  |  |  |  |  |  |  |  |  |  |  |  |  |  |  |  |  |  |  |  |  |  |  |  |  |  |  |
| Endline survey |  |  |  |  |  |  |  |  |  |  |  |  |  |  |  |  |  |  |  |  |  |  |  |  |  |  |  |  |  |  |  |
| PCR analysis of endline survey samples |  |  |  |  |  |  |  |  |  |  |  |  |  |  |  |  |  |  |  |  |  |  |  |  |  |  |  |  |  |  |  |
| Endline Survey data process and cleaning |  |  |  |  |  |  |  |  |  |  |  |  |  |  |  |  |  |  |  |  |  |  |  |  |  |  |  |  |  |  |  |
| Evaluation analysis |  |  |  |  |  |  |  |  |  |  |  |  |  |  |  |  |  |  |  |  |  |  |  |  |  |  |  |  |  |  |  |
| Final dissemination of results |  |  |  |  |  |  |  |  |  |  |  |  |  |  |  |  |  |  |  |  |  |  |  |  |  |  |  |  |  |  |  |

# References

1. National Malaria Strategic Plan 2011-2015. *NMCC* (2011).

2. Zambia, M. of H. Government of the Republic of *Zambia National Malaria Indicator Survey 2015*. (Government of the Republic of Zambia: 2015).

3. Zambia, M. of H. Government of the Republic of *Zambia National Malaria Indicator Survey 2012*. (Government of the Republic of Zambia: 2012).

4. Zambia, M. of H. Government of the Republic of *Zambia National Malaria Indicator Survey 2010*. (2010).at <http://www.nmcc.org.zm/files/FullReportZambiaMIS2010_001.pdf>

5. Zambia, M. of H. Government of the Republic of *Zambia National Malaria Indicator Survey 2008*. (2008).

6. Zambia, M. of H. Government of the Republic of *Zambia National Malaria Indicator Survey 2006*. (2006).

7. Johnston, G. L., Gething, P. W., Hay, S. I., Smith, D. L. & Fidock, D. A. Modeling within-host effects of drugs on Plasmodium falciparum transmission and prospects for malaria elimination. *PLoS Comput Biol* **10**, e1003434 (2014).

8. Moonen, B. *et al.* Operational strategies to achieve and maintain malaria elimination. *Lancet* **376**, 1592–1603 (2010).

9. Stresman, G. H. *et al.* A method of active case detection to target reservoirs of asymptomatic malaria and gametocyte carriers in a rural area in Southern Province, Zambia. *Malar J* **9**, 265 (2010).

10. Bousema, T. *et al.* Hitting hotspots: spatial targeting of malaria for control and elimination. *PLoS Med* **9**, e1001165 (2012).

11. Seidlein, L. von & Greenwood, B. M. Mass administrations of antimalarial drugs. *Trends Parasitol* **19**, 452–460 (2003).

12. Kaneko, A. *et al.* Malaria eradication on islands. *Lancet* **356**, 1560–1564 (2000).

13. Hsiang, M. S. *et al.* Mass drug administration for the control and elimination of Plasmodium vivax malaria: an ecological study from Jiangsu province, China. *Malar J* **12**, 383 (2013).

14. Poirot, E. *et al.* Mass drug administration for malaria. *Cochrane Database Syst Rev* **12**, CD008846 (2013).

15. Okell, L. C., Ghani, A. C., Lyons, E. & Drakeley, C. J. Submicroscopic infection in Plasmodium falciparum-endemic populations: a systematic review and meta-analysis. *J Infect Dis* **200**, 1509–1517 (2009).

16. Okell, L. C. *et al.* Factors determining the occurrence of submicroscopic malaria infections and their relevance for control. *Nat Commun* **3**, 1237 (2012).

17. Mouatcho, J. C. & Goldring, J. P. D. Malaria rapid diagnostic tests: challenges and prospects. *J Med Microbiol* **62**, 1491–1505 (2013).

18. Okell, L. C. *et al.* The potential contribution of mass treatment to the control of Plasmodium falciparum malaria. *PLoS One* **6**, e20179 (2011).

19. Davis, T. M., Karunajeewa, H. A. & Ilett, K. F. Artemisinin-based combination therapies for uncomplicated malaria. *Medical Journal of Australia* **182**, 181 (2005).

20. Ezzet, F., Vugt, M. van, Nosten, F., Looareesuwan, S. & White, N. J. Pharmacokinetics and pharmacodynamics of lumefantrine (benflumetol) in acute falciparum malaria. *Antimicrob Agents Chemother* **44**, 697–704 (2000).

21. Davis, T. M., Hung, T.-Y., Sim, K., Karunajeewa, H. A. & Ilett, K. F. Piperaquine: a resurgent antimalarial drug. *Drugs* **65**, 75–87 (2005).

22. Daniels, R. *et al.* Genetic Surveillance Detects Both Clonal and Epidemic Transmission of Malaria following Enhanced Intervention in Senegal. *PLoS One* **8**, e60780 (2013).

23. Drakeley, C. & Cook, J. Chapter 5. Potential contribution of sero-epidemiological analysis for monitoring malaria control and elimination: historical and current perspectives. *Adv Parasitol* **69**, 299–352 (2009).

24. Cook, J. *et al.* Sero-epidemiological evaluation of changes in Plasmodium falciparum and Plasmodium vivax transmission patterns over the rainy season in Cambodia. *Malar J* **11**, 86 (2012).

25. Drakeley, C. J. *et al.* Estimating medium- and long-term trends in malaria transmission by using serological markers of malaria exposure. *Proc Natl Acad Sci U S A* **102**, 5108–5113 (2005).

26. Drame, P. M. *et al.* IgG responses to the gSG6-P1 salivary peptide for evaluating human exposure to Anopheles bites in urban areas of Dakar region, Sénégal. *Malar J* **11**, 72 (2012).

27. Wu, L. *et al.* Comparison of diagnostics for the detection of asymptomatic Plasmodium falciparum infections to inform control and elimination strategies. *Nature* **528**, S86–S93 (2015).

28. Slater, H. C. *et al.* Assessing the impact of next-generation rapid diagnostic tests on Plasmodium falciparum malaria elimination strategies. *Nature* **528**, 94–101 (2015).

29. Hemingway, J. *et al.* Tools and Strategies for Malaria Control and Elimination: What Do We Need to Achieve a Grand Convergence in Malaria? *PLoS biology* **14**, e1002380 (2016).

30. Keating, G. M. Dihydroartemisinin/Piperaquine: a review of its use in the treatment of uncomplicated Plasmodium falciparum malaria. *Drugs* **72**, 937–961 (2012).

31. Myint, H. Y., Ashley, E. A., Day, N. P. J., Nosten, F. & White, N. J. Efficacy and safety of dihydroartemisinin-piperaquine. *Transactions of the Royal Society of Tropical Medicine and Hygiene* **101**, 858–866 (2007).

32. Bigira, V. *et al.* Protective efficacy and safety of three antimalarial regimens for the prevention of malaria in young ugandan children: a randomized controlled trial. *PLoS Med* **11**, e1001689 (2014).

33. Lwin, K. M. *et al.* Randomized, double-blind, placebo-controlled trial of monthly versus bimonthly dihydroartemisinin-piperaquine chemoprevention in adults at high risk of malaria. *Antimicrobial agents and chemotherapy* **56**, 1571–1577 (2012).

34. Guillot, G., Mortier, F. & Estoup, A. GENELAND: a computer package for landscape genetics. *Molecular Ecology Notes* **5**, 712–715 (2005).

35. R: A Language and Environment for Statistical Computing. (2010).at <http://www.R-project.org>

36. Aimone, A. M., Perumal, N. & Cole, D. C. A systematic review of the application and utility of geographical information systems for exploring disease-disease relationships in paediatric global health research: the case of anaemia and malaria. *Int J Health Geogr* **12**, 1 (2013).

37. Dambach, P. *et al.* Utilization of combined remote sensing techniques to detect environmental variables influencing malaria vector densities in rural West Africa. *Int J Health Geogr* **11**, 8 (2012).

38. Clennon, J. A., Kamanga, A., Musapa, M., Shiff, C. & Glass, G. E. Identifying malaria vector breeding habitats with remote sensing data and terrain-based landscape indices in Zambia. *Int J Health Geogr* **9**, 58 (2010).

39. Nmor, J. C. *et al.* Topographic models for predicting malaria vector breeding habitats: potential tools for vector control managers. *Parasit Vectors* **6**, 14 (2013).

40. Achee, N. L. *et al.* Use of remote sensing and geographic information systems to predict locations of Anopheles darlingi-positive breeding sites within the Sibun River in Belize, Central America. *Journal of medical entomology* **43**, 382–392 (2006).

41. Guillot, G., Estoup, A., Mortier, F. & Cosson, J. F. A spatial statistical model for landscape genetics. *Genetics* **170**, 1261–1280 (2005).

42. Davies, T. M. & Hazelton, M. L. Adaptive kernel estimation of spatial relative risk. *Statistics in Medicine* **29**, 2423–2437 (2010).

43. Hayes, R. & Bennett, S. Simple sample size calculation for cluster-randomized trials. *International journal of epidemiology* **28**, 319–326 (1999).

44. Hofmann, N. *et al.* Ultra-Sensitive Detection of Plasmodium falciparum by Amplification of Multi-Copy Subtelomeric Targets. *PLoS Med* **12**, e1001788 (2015).

45. Lucchi, N. W. *et al.* Molecular diagnosis of malaria by photo-induced electron transfer fluorogenic primers: PET-PCR. *PloS one* **8**, e56677 (2013).

46. Daniels, R. *et al.* A general SNP-based molecular barcode for Plasmodium falciparum identification and tracking. *Malar J* **7**, 223 (2008).

47. Zani, B., Gathu, M., Donegan, S., Olliaro, P. L. & Sinclair, D. Dihydroartemisinin-piperaquine for treating uncomplicated Plasmodium falciparum malaria. *Cochrane Database Syst Rev* **1**, CD010927 (2014).

# Appendices

Appendix 1 Step D Protocol: Current RFTAT intervention in Southern Province v1

Appendix 2 Active Infection Detection Protocol: Current RFTAT intervention in Lusaka v1

Appendix 3 Consent and Information Sheet for Household Survey: Endline surveys v4

Appendix 4 MIS Questionnaire for Parasite Evaluation: Endline survey questionnaire v2

Appendix 5 Oral Assent for Children 6-17 years for DHAP administration v2

Appendix 6 RFTAT Questionnaire: Research responses v2

Appendix 7 RFDA Questionnaire: Research responses v2

Appendix 8 Longitudinal Study Consent Form: Research responses on days 0, 3, 30 and 90 v3

Appendix 9 3-day post Questionnaire: Research responses v2

Appendix 10 30-day and 90-day post Questionnaire: Research responses v2

Appendix 11 Consent and information sheet for administration of DHAP (RFDA Arm only) v6

Appendix 12 Routine Step B and Step D Data elements collected v1

Appendix 13 Adverse event reporting form v2

Appendix 14 Adverse event investigation form v2

Appendix 15 Register of recording adverse events v1

Appendix 16 Oral Assent for Children 6-17 years for malaria blood test v2

Appendix 17 CHW register for RFTAT v1

Appendix 18 CHW register for RFDA v1

Appendix 19 Oral Assent for Children 6-15 years for malaria survey
