# Supplementary material for: Reactive focal drug administration associated with decreased malaria transmission in an elimination setting: Serological evidence from the cluster-randomized CoRE study
Source: PLOS Glob Public Health. 2022 Dec 5;2(12):e0001295. doi: 10.1371/journal.pgph.0001295 (PMC10021141; doi:10.1371/journal.pgph.0001295)
Supplement: S2 File — (PDF) [file pgph.0001295.s013.pdf]

THE UNIVERSITY OF ZAMBIA

BIOMEDICAL RESEARCH ETHICS COMMITTEE

Telephone: 260-1-256067
Telegrams: UNZA, LUSAKA
Telex: UNZALU ZA 44370
Fax: + 260-1-250753
E-mail: unzarec@unza.zm

Ridgeway Campus
P.O. Box 50110
Lusaka, Zambia

Assurance No. FWA00000338
IRB00001131 of IORG0000774

13th February, 2015.

Our Ref: 011-10-14.

Dr. Daniel Bridges,
AKROS,
Unit 5, Cresta Golfview Grounds,
Great East Road,
Lusaka.

Dear Dr. Bridges,

RE: RESUBMITTED RESEARCH PROPOSAL: **"COMMUNITY-LED RESPONSES FOR ELIMINATION (CORE): A CLUSTER RANDOMIZED CONTROLLED TRIAL OF REACTIVE CASE DETECTION VERSUS REACTIVE DRUG ADMINISTRATION IN MALARIA ELIMINATION AREAS" (REF. No. 011-10-14)**

The above-mentioned research proposal was presented to the Biomedical Research Ethics Committee on 7th January, 2015. The proposal is approved.

CONDITIONS:

- This approval is based strictly on your submitted proposal. Should there be need for you to modify or change the study design or methodology, you will need to seek clearance from the Research Ethics Committee.
- If you have need for further clarification please consult this office. Please note that it is mandatory that you submit a detailed progress report of your study to this Committee every six months and a final copy of your report at the end of the study.
- Any serious adverse events must be reported at once to this Committee.
- Please note that when your approval expires you may need to request for renewal. The request should be accompanied by a Progress Report (Progress Report Forms can be obtained from the Secretariat).
- **Ensure that a final copy of the results is submitted to this Committee.**

Yours sincerely,

M.M.Mbewe (mrs)
CHAIRPERSON

Date of approval: 13th February, 2015. **Date of expiry:** 12th February, 2016.

WIRB
Global Leader | Proven Expert

Western Institutional Review Board®
1019 39th Avenue SE Suite 120 | Puyallup, WA 98374-2115
Office: (360) 252-2500 | **Toll Free**: (800) 562-4789
www.wirb.com

Certificate of Approval

THE FOLLOWING WERE APPROVED

INVESTIGATOR: John M. Miller PhD, MPH
Daniel Bridges PhD, MSc
National Malaria Control Centre
Great East Road
Lusaka, Lusaka
Zambia

BOARD ACTION DATE: 09/14/2015
PANEL: 3
STUDY APPROVAL EXPIRES: 09/14/2016
STUDY NUM: 1155095
WIRB PRO NUM: 20150932
ONLINE TRACKING: 11-
INVEST NUM: 190668
WO NUM: 1-884991-1
CONTINUING REVIEW: Annually
SITE STATUS REPORTING: Annually
INST. NUM: 726303-1

SPONSOR: PATH
PROTOCOL NUM: None
AMD. PRO. NUM:
TITLE:
Community-led Responses for Elimination (CoRE): A cluster randomized controlled trial of reactive case detection versus reactive drug administration in malaria elimination areas

APPROVAL INCLUDES:
Investigator
Investigator
30-day and 90-day post reaction questionnaire #13116656.0 - As Submitted
3-day post reaction questionnaire #13116655.0 - As Submitted
Protocol (04-27-2015) Version 5
Reactive focal drug administration questionnaire #13116653.0 - As Submitted
Reactive focal test and treat questionnaire #13116652.0 - As Submitted
Revised Protocol (06-18-2015) Version 6
Revised Protocol (09-02-2015) Version 7
Zambia Malaria Indicator Survey #13116650.0 - As Submitted
Assent Information Sheet - DHAP Administration [S0]
Assent Information Sheet [S0]
Consent Form - Administration of DHAP [S0]
Consent Form - Household Survey [S0]
Consent Form - Reactive Response Surveys [S0]

WIRB APPROVAL IS GRANTED SUBJECT TO:
The Board made the following assent requirements: The investigator will determine whether assent and its documentation are required for each subject on a case-by-case basis. Documentation of the capability determination and the assent if appropriate are required using the assent section on the consent form. Individual institutional requirements are captured on the consent and assent documents.

The Board made the following assent requirements: Assent is not required for children under 6 years due to their limited capability. Verbal assent is required for children over 6 but under 18 years using the assent section on the parental permission form. Individual institutional requirements are captured on the consent and assent documents.

WIRB HAS APPROVED THE FOLLOWING LOCATIONS TO BE USED IN THE RESEARCH:
Ministry of Health (Southern Province), Provincial Medical Office, Choma, Southern Province Zambia

IF YOU HAVE ANY QUESTIONS, CONTACT WIRB AT 1-800-562-4789
This is to certify that the information contained herein is true and correct as reflected in the records of the Western Institutional Review Board (WIRB), OHRP/FDA parent organization number IORG 0000432, IRB registration number IRB00000533. WE CERTIFY THAT WIRB IS IN FULL COMPLIANCE WITH GOOD CLINICAL PRACTICES AS DEFINED UNDER THE U.S. FOOD AND DRUG ADMINISTRATION (FDA) REGULATIONS, U.S. DEPARTMENT OF HEALTH AND HUMAN SERVICES (HHS) REGULATIONS, AND THE INTERNATIONAL CONFERENCE ON HARMONISATION (ICH) GUIDELINES.

Full AAHRPP Accreditation

Copyright © 2013 Western Institutional Review Board, Inc. All rights reserved.

If the PI has an obligation to use another IRB for any site listed above and has not submitted a written statement from the other IRB acknowledging WIRB's review of this research, please contact WIRB's Client Services department.

ALL WIRB APPROVED INVESTIGATORS MUST COMPLY WITH THE FOLLOWING:

1. Conduct the research in accordance with the protocol, applicable laws and regulations, and the principles of research ethics as set forth in the Belmont Report.

2. Although a participant is not obliged to give his or her reasons for withdrawing prematurely from the clinical trial, the investigator should make a reasonable effort to ascertain the reason, while fully respecting the participant's rights.

3. Unless consent has been waived, conduct the informed consent process without coercion or undue influence, and provide the potential subject sufficient opportunity to consider whether or not to participate. (Due to the unique circumstances of research conducted at international sites outside the United States and Canada, when there is a local IRB and WIRB approved materials are reviewed by the local IRB and translated into the local language, the following requirements regarding consent forms bearing the WIRB approval stamp and regarding certification of translations are not applicable.)
   a. Use only the most current consent form bearing the WIRB "APPROVED" stamp.
   b. Provide non-English speaking subjects with a certified translation of the approved consent form in the subject's first language. The translation must be approved by WIRB unless other arrangements have been made and approved by WIRB.
   c. Obtain pre-approval from WIRB for use of recruitment materials and other materials provided to subjects.

4. Enrollment of limited readers and non-readers: unless consent has been waived or the protocol excludes enrollment of limited readers or non-readers, involve an impartial witness in the consent process when enrolling limited or non-readers and document the participation of the impartial witness using the designated signature lines on the WIRB-approved consent form. In the absence of designated signature lines, download the WIRB standard impartial witness form from www.wirb.com.

5. Obtain pre-approval from WIRB for changes in research.

6. Obtain pre-approval from WIRB for planned deviations and changes in research activity as follows:
   - If the research is federally funded, conducted under an FWA, or is a clinical investigation of a drug or biologic, then all planned protocol deviations must be submitted to WIRB for review and approval prior to implementation except where necessary to eliminate apparent immediate hazards to the human subjects [(DHHS 45 CFR § 46.103(b)(4); (FDA 21 CFR § 56.108(a)(4); ICH 3.3.7].
   - However, if the research is a clinical investigation of a device and the research is not federally funded and not conducted under an FWA, then only planned protocol deviations that may adversely affect the rights, safety or welfare of subjects or the integrity of the research data should be submitted to WIRB for review and approval prior to implementation except where necessary to eliminate apparent immediate hazards to the human subjects [(DHHS 45 CFR § 46.103(b)(4); (FDA 21 CFR § 56.108(a)(4); ICH 3.3.7].

   The reason for these different requirements regarding planned protocol deviations is that the Office for Human Research Protections (OHRP) and the Food and Drug Administration (FDA) drug and biologic divisions have adopted the regulatory interpretation that every planned protocol deviation is a change in research that needs prior IRB review and approval before implementation; however, the FDA device division operates under a distinct regulation (See 21 CFR 812.150(a)(4).

   Deviations necessary to eliminate apparent immediate hazards to the human subjects should be reported within 10 days.

7. Report the following information items to the IRB within 5 days:
   a. New or increased risk
   b. Protocol deviation that harmed a subject or placed subject at risk of harm
   c. Protocol deviation made without prior IRB approval to eliminate an immediate hazard to a subject
   d. Audit, inspection, or inquiry by a federal agency
   e. Written reports of federal agencies (e.g., FDA Form 483)
   f. Allegation of Noncompliance or Finding of Noncompliance
   g. Breach of confidentiality
   h. Unresolved subject complaint
   i. Suspension or premature termination by the sponsor, investigator, or institution
   j. Incarceration of a subject in a research study not approved to involve prisoners
   k. Adverse events or IND safety reports that require a change to the protocol or consent
   l. State medical board actions
   m. Unanticipated adverse device effect
   n. Information where the sponsor requires prompt reporting to the IRB

 Copyright © 2011 Western Institutional Review Board, Inc. All rights reserved.

Information not listed above does not require prompt reporting to WIRB.

Please go to www.wirb.com for complete definitions and forms for reporting.

8. Provide reports to WIRB concerning the progress of the research, when requested.

9. Ensure that prior to performing study-related duties, each member of the research study team has had training in the protection of human subjects appropriate to the processes required in the approved protocol.

Federal regulations require that WIRB conduct continuing review of approved research. You will receive Continuing Review Report forms from WIRB. These reports must be returned even though your study may not have started.

DISTRIBUTION OF COPIES:
Contact, Company
Elizabeth Trias MA, PATH
John M Miller PhD, MPH, PATH
Richard Steketee, PATH
Daniel Bridges PhD, MSc, Akros

Copyright © 2011 Western Institutional Review Board, Inc. All rights reserved.

All correspondence should be
addressed to the Director General

In reply, please quote

DMS/7/9/22/CT/052

ZAMBIA MEDICINES REGULATORY AUTHORITY

5th October, 2015

Daniel Bridges
Unit 5 Cresta Golfview Grounds
Great East Road
Lusaka

Email: dbridges@akros.com

Dear Sir/Madam

RE: COMMUNITY – LED RESPONSES FOR ELIMINATION (CoRE): A CLUSTER RANDOMISED CONTROLLED TRIAL OF REACTIVE CASE DETECTION VERSUS REACTIVE DRUG ADMINISTRATION IN MALARIA ELIMINATION

Reference is made to your letter dated 4th September, 2015 regarding the above stated clinical study.

We acknowledge receipt of the updated protocol, patient information leaflet and consent forms. We wish to advise that the Zambia Medicines Regulatory Authority (ZAMRA) considered the submitted additional information and **approved** the conduct of the following study with ZAMRA clinical trial number and protocol number as indicated below:

No.	Name of Clinical Trial	Clinical Trial No.	Protocol No.	Version
1.	**Community- led Responses for elimination (CoRE):** A cluster, randomised controlled trial of reactive case detection versus reactive drug administration in malaria elimination areas	CT 052	1	5

We also wish to advise that you are required to provide periodic updates on the study and report any adverse events that may occur during the study. Furthermore, ZAMRA will carry out clinical trial site inspections as it may deem necessary.

Should you have any questions, please do not hesitate to contact the Secretariat.

Yours faithfully,
for/Zambia Medicines Regulatory Authority

E Mwape (Ms)
DIRECTOR GENERAL

Head Office
Plot No: 6903, TuletekaRoad/ Off Makishi Road
P.O. Box 31890 Lusaka, ZAMBIA
Tel: +260 211 220429, Telefax: +260 211 238458
E-mail: pharmacy@zamra.co.zm

Ndola Office
No: 41 Kafironda Drive, Itawa
P.O. Box 70876
Telefax: +260 212 610522

Website: www.zamra.co.zm

Report Adverse Reactions to:
Pharmacovigilance Unit, Lusaka
Tel: +260 211 220088 / 220089 / 220109
E-mail: pharmacy@zamra.co.zm
