# Supplementary material for: Reactive focal drug administration associated with decreased malaria transmission in an elimination setting: Serological evidence from the cluster-randomized CoRE study
Source: PLOS Glob Public Health. 2022 Dec 5;2(12):e0001295. doi: 10.1371/journal.pgph.0001295 (PMC10021141; doi:10.1371/journal.pgph.0001295)
Supplement: Attachment — Submitted filename: Response to reviewers.docx [file pgph.0001295.s015.docx]

Editorial Office

PLOS Global Public Health

29^th^ September 2022

Our responses to the journal requirements and reviewers’ comments can be found below:

# Journal Requirements

1. Please include additional information regarding the survey or questionnaire used in the study and ensure that you have provided sufficient details that others could replicate the analyses. For instance, if you developed the survey or questionnaire as part of this study and it is not under a copyright more restrictive than CC-BY, please include a copy, in both the original language and English, as Supporting Information. If the questionnaire is published, please provide a citation to the questionnaire.

We used the survey developed by the National Malaria Elimination Center, Zambia for the 2015 Malaria Indicator Survey. We adjusted the methods to reflect this including the citation for the report of the survey.

2. In your Methods section, please provide additional information about the participant recruitment method and the demographic details of your participants. Please ensure you have provided sufficient details to replicate the analyses such as: 
a) a table of relevant demographic details, 
b) how participants were recruited.

a) We have no demographic data on the participants recruited during the routine interventions that were performed by the CHWs. We do have demographic data for individuals recruited for longer-term follow-ups on days 30 and 90, we have included these details as a supplemental table (S10 Table). We also have included another supplemental table (S11 Table) with demographic information on individuals who participated in the post-only evaluation survey.

b) We have revised the methods section to clarify how participants were recruited and to what aspects of the study they were involved in (the intervention or the evaluation). The first subheading under the methods section is now “Intervention and participants”, and we added an “Evaluation Study Design” subheading directly after. We now describe the recruitment of intervention participants in the “Intervention and Participants” section. Specifically, they were recruited based on living within a 140m radius of the incident malaria case. We now describe the recruitment of participants in the cross-sectional survey in the Data Sources section. Specifically, they were recruited from randomly selected households identified from satellite enumerated imagery.

3. Please provide additional details regarding participant consent. In the ethics statement in the Methods, please ensure that you have specified what type of consent you obtained (for instance, written or verbal, and if verbal, how it was documented and witnessed). If your study included minors, state whether you obtained consent from parents or guardians.

We have revised the methods to describe the consent process more clearly for each of the different stages.

4. Please amend your detailed Financial Disclosure statement. This is published with the article. It must therefore be completed in full sentences and contain the exact wording you wish to be published.

State what role the funders took in the study. If the funders had no role in your study, please state: “The funders had no role in study design, data collection and analysis, decision to publish, or preparation of the manuscript.”

Thank you for this addition – we have included a ‘Funding’ section in the manuscript that outlines our position.

5. Please provide separate figure files in .tif or .eps format and ensure that all files are under our size limit of 10MB.

For more information about how to convert your figure files please see our guidelines: <https://journals.plos.org/globalpublichealth/s/figures>

All figure files have been checked with PACE and conform to the journal requirements.

# Reviewer Comments

## Reviewer #1:

Thank you very much for sending me this paper for review. The investigators studied two interventions in southern Zambia:
a) Reactive presumptive treatment
b) Reactive test and treat

Both intervention are timely and relevant as national malaria control programmes aiming for malaria elimination.

We thank the reviewer. One point of clarification, reactive test and treat is the standard of care in Zambia and as such is the control arm. We have clarified this in the methods section.

The authors found at the end of the study period the percentage of seropositive was lower in presumptive treatment group (a) than in the test in treat group (b). This result makes intuitively sense as the authors explain. The diagnostic tools to detect very low density, subclinical infections in field settings are not yet available. Treating everybody within a certain radius around the index patients is logistically easier and likely to be more effective. The investigators did not find a difference in parasite prevalence or incidence which are the much more frequently used outcomes of similar, large programmatic malaria intervention studies. Serology is a complex endpoint as it includes a number of antibodies and requires sets of derivations to determine who is infected and who is not. When incidence and prevalence estimates suggest that there is no difference between the two interventions it is courageous to say that the rather derivative serology results indicate the true impact of the intervention and incidence and prevalence estimates are underpowered hence as the authors suggest.

We knew at the outset that parasite prevalence in our achievable sample size would be underpowered. Hence, we designed the trial around the primary outcome of seroprevalence. Serology has been used primarily in malaria elimination settings, although perhaps ours is one of the first to apply it to intervention trials. Regardless, powering the study to malaria parasite prevalence in this setting was a non-starter due to the unattainable sample size required.

There are two major design flaws which will make it difficult to convince readers:
There is no control group - which did not receive an intervention. We can therefore say that one intervention is as good or better than the other but we do not know the absolute impact on malaria. The fact that the malaria burden decreased over the study period suggests that the interventions made a positive impact but alternatively this decrease could be caused by other factors than the study. As the authors write "This decline was observed across Southern Province, Zambia and coincided with lower-than-average rainfall." Suggesting a secular event independent of the study interventions.

We do not consider this a design flaw, but rather a study limitation. But it is a limitation enforced by ethics rather than scientific design. RFTAT is the standard of care in Zambia, and hence it is the control arm. We have clarified this in the methods. We compared the two interventions in a superiority trial, and the results are as we report.

Second the investigators did not estimate the baseline serostatus in their study population. The authors may be right that the presumptive treatment reduced transmission more than the test and treat intervention did. Alternatively, the seroprevalence in the test and treat clusters was already higher at baseline. The authors provide indirect, unvalidated evidence that this is not the case as there was a difference in seroprevalence by age group. With all due respect I find this speculative and far from convincing. To make the serology data convincing the authors would have to show that the baseline seroprevalence was similar in both study arms.

We thank the reviewer and agree that a baseline seroprevalence would have been ideal. However, ethically we could not collect blood samples in a survey without providing a benefit to the participant. In this case, we provided malaria testing (and treatment for positive individuals) for all participants. Such a survey essentially creates a mass test and treat event. We therefore purposely designed the study without a baseline to avoid introducing an additional factor that could bias the outcomes. To control for a lack of a baseline, we instead relied on the seroprevalence by age group. We understand the reviewer’s hesitancy in this outcome and have included this in the limitations. We also note that seroprevalence by age group has been used to understand historical changes in malaria transmission. Specifically, we included the following text and have sought to highlight these issues in the ‘Study Limitations’ section:

“The post-only comparison of sero-prevalence is limited in that there was no pre-intervention estimate of seroprevalence. We opted to exclude the pre-intervention estimate of seroprevalence in order to ensure intervention fidelity. Ethically we would be required to treat every malaria infection found during a baseline survey, making the baseline survey itself a mass testing and treatment event, which does have an effect on malaria transmission [31]. It could be that malaria transmission was inherently lower in the RFDA group than the RFTAT group independent of the intervention. We have attempted to account for the lack of a pre-intervention baseline by estimating seroprevalence by age group.”

“The use of seroprevalence by age group as an historical indicator of transmission has been used in studies of malaria elimination previously, but to our knowledge we are the first to use these measures in an intervention trial.”

It is probably impossible to address these flaws retrospectively. The only appropriate way to deal with this problem is to address these challenges clearly and honestly and then discuss the implications of these major limitations. The project must have been a major effort in terms of human and financial resources. The data are interesting and should be published but the data don't support the conclusion "These results provide robust confidence that the RFDA intervention reduced malaria transmission …" the seroprevalence estimates are flawed without a baseline estimate and the more direct, easy to interpret incidence and prevalence data suggest there is no difference between the treatment arms. The authors have to make this clear throughout the paper starting with the title.

We disagree with the reviewer that these are design flaws, as they were intentional. They are limitations, however. As such we have revised the title (page 1) and our conclusion (page 17, line 20) accordingly. In our view parasite prevalence is not a useful indicator in malaria elimination settings, and so we are left with discordant health facility incidence and population-representative seroprevalence results. Given the plethora of limitations with health facility incidence we put additional weight on the population-representative seroprevalence results.

In the discussion the authors say "PCR identified a very limited number of infections (n = 16, 0.25%), and not enough to make a meaningful comparison between the two arms..." suggesting that if only more case would have been detected the desired outcome would have been reached. This conclusion is entirely inappropriate. The authors wish that their work made an impact and if only their study had more power, they could have shown such an impact. While such an interpretation is entirely understandable there is the alternatively interpretation that their intervention made no impact. They can't disprove the null hypothesis and therefore have to accept the null hypothesis. The statement "It is possible that increasing the survey sample could have generated enough data to compare the arms, however the increase, and therefore cost, would have been dramatic and likely required a complete census rather than survey." Sorry, but you do not have the evidence to support this statement. It suggests that the authors are not familiar with the basic elements of hypothesis testing. We always hope that our interventions work but if there is no evidence, we have to accept that we failed to disprove the null hypothesis as disappointing and painful as it may be.

We designed the study around seroprevalence rather than parasite prevalence. The malaria parasite prevalence in this study area made it essentially impossible to design a study using this measure. First, we desired to avoid a mass testing and treatment intervention as part of a baseline survey, a process that would have altered malaria transmission due to the ethically required treatment of positive individuals. Thus, we would have been left with either a post-only malaria parasite prevalence measure or a treatment effect in the context of a mass testing and treatment event.

We agree with the reviewer that we need to accept the results as they are and have attempted to do so. We place more confidence in the population-representative seroprevalence survey than in the health facility incidence, but we place no value in malaria parasite prevalence in this context.

For the reviewers’ benefit, a quick calculation of power using Hayes et al. for parasite prevalence of 0.5% reduced to 0.25% and assuming a coefficient of variation of 0.25 (standard when unknown). Sampling 120 children per health facility would require 82 health facilities per trial arm – 164 in total, more than exist in the elimination areas of Southern Province. Increasing the sample size per health facility to 500 children would only require 23 health facilities per trial arm – 46 in total, but many catchments would likely not have 500 children forcing us to increase the age range to include adults. Finally increasing the sampling to such a large proportion of the population would create an even greater mass testing and treatment event, and so again we would be left with a post-only comparison of prevalence. (We cannot ethically test someone for malaria and not offer treatment if positive).

If the authors decide to revise their paper and address these major concerns they may wish also to address the following points:

The consort checklist for individually randomised trials is not very appropriate for cluster randomised trials. Please consider complying with the checklist for cluster randomised trials?

Thank you for bringing this to our attention. We have switched to the checklist for cluster randomized trials.

Figure 1, the consort chart illustrating the patient assembly is not very helpful. It would be much more interesting to see how many participants were recruited in each study arm and how many samples were analysed in the serology study. This is nicely explained in the consort checklist for cluster randomised trials.

Thank you for this recommendation we have updated Figure 1, including the avg cluster size and variance. Randomisation was performed for the entire community at each health facility (cluster). The number of individuals enrolled in each arm is given in Table 1. The number of samples analysed in the endline serology study is mentioned in the Results section under the Cross-sectional survey sub-heading.

At one point the authors refer to the "intervention arm". Since we are dealing with two arms each receiving different interventions this statement is not helpful.

We have clarified in the manuscript that RFTAT is the standard of care, and hence is the control arm.

The figures have no embedded legends it is difficult to guess what the colours mean. What is shown along the x-axis in Figure 4?

The x-axis represents individual health facilities. Each panel of the figure orders the health facility from maximum to minimum seropositivity observed (left to right). We have sought to clarify this in the figure caption and have included the colours in the figure caption.

The imbalance of adverse events in the treatment arms is surprising. It would be interesting to know how the investigators explain this finding.

We believe there are a few reasons for this. Firstly, significantly less antimalarials were disbursed in the control RFTAT arm (90 courses) compared to the intervention RFDA arm (1,775 courses - Table 1). Secondly, because RFTAT with AL is the standard of care we did not consent anyone into the receipt of AL during RFTAT (unlike RFDA and DHAP). Therefore, when we asked for adverse events in the RFTAT arm the participants were not primed in the same way that they were in the RFDA arm. Finally, AL is widely available and has been used for almost 20 decades in country. It is well-known and accepted. We believe that recipients in the control arm were therefore less likely to recognize / report any adverse events.

Overall, it does not help that the preparation of the manuscript is rather sloppy. Error warnings "Error! Reference source not found." appear repeatedly in the manuscript which should have been corrected before approving the submission.

Our apologies for these oversights we have now fixed these errors.

## Reviewer #2:

Please indicate the major vector that was transmitting malaria. The flight range of this vector will determine the radius of the area of reactive case detection with reference to the index case. For example the flight range of An funestus is much smaller that that of An gambiae s.l.

We thank the reviewer for the helpful suggestions. We have included this information in the Methods section (page 4, line 20-22), specifically the following sentence: “*Anopheles arabiensis* is considered to be the primary vector in the study area [1], although numerous other vectors do contribute to transmission [2].”

It would be useful to indicate the half life of the short live verses the long lived antibodie in the discussion. The authors should also discuss the rate of decay of the short-lived antibodies during chemotherapy with both drug treatment regiments.

Calculating multiple antibody decay rates is not a trivial undertaking as it would require longitudinal sampling of infected individuals over a significant time scale (years). A study identifying serological markers that accurately identified historical infections has been performed by Helb et al 2015, many of which are included in this study and was able to relatively accurately predict infections over a one year period. Drakeley et al 2005 estimated a seroreversion rate for a long-term marker at ~0.014 or ~70 years. Even taking a drastically more conservative estimate would suggest seroreversion is insignificant over the timeline used in this study. Of course, due to the high heterogeneity that different populations and individuals show, confirmatory studies may need to be performed in the population of interest rather than using generalizable data from other populations. As a result, for this study it is difficult to be any more specific than identifying some responses as short-lived (weeks to months) and others as long-lived (years to decades). We don’t believe that chemotherapy would affect antibody decay rates, except in so much as it may arrest the development of a more robust immune response.

As an alternative, would it have been feasible to use the rate of short lived antibody decay as a measure of diminishing transmission ? See Ototo 2011

If we understand this correctly, the reviewer is suggesting that antibody decay rates correlate to transmission intensity. This may be true when there are large changes in transmission intensity e.g., from high transmission intensities where individuals experience hyperinfections compared to low transmission intensities where infections are isolated and resolve in isolation. However, in elimination settings, moving from low to zero seems unlikely to change antibody decay rates. As with the previous point, generating a decay rate would require longitudinal sampling for each participant, which we did not do. As regards the Ototo 2011 reference, this paper used a qualitative binary outcome rapid diagnostic test for both CSP and MSP antigens. The authors identified CSP-MSP positivity as an early predictor for sporozoite positivity and suggest this could be a useful indicator of the onset of hyper-transmission during climatic-driven outbreaks. We are unsure how this would apply to the setting the CoRE study was applied to.

## Reviewer #3:

This manuscript deals with a very important problem when striving to malaria elimination: how to reduce malaria and evaluate progress in areas with a low level of malaria. They present results from a trial that contrasts a reactive case detection strategy with a reactive case and presumptive treat scenario, with innovative tools to evaluate progress in both scenarios. The trial was entered in a registry before the start of the study, and protocol, and data have been made available to the reviewers, enhancing full transparency. However, the manuscript can be improved with a better description of methodology.

We thank the reviewer for the suggested revisions. We have revised the methodology to increase clarity as best we can. We are happy to further revise with any specific issues.

Abstract
"Comparing long-term serological markers, we found a 30% (95% CI = 21-37%) reduction in seropositivity for the RFDA intervention using a difference in diffeerences approach incorporating serological positivity and age". I would prefer if the authors stick to the under-fives here for what was reported in the result section: "while under 5's in the RFDA arm were 19% (95% CI = 4-32%) less likely to test positive than under 5s in the RFTAT arm." Table 2 does not show a difference by treatment overall.

We actually recorded the wrong parameter here in the abstract and discussion. We should have been reporting the interaction term from table 2 (19% reduction), which provides the difference in differences result. We observed higher seropositivity in the RFTAT arm among older individuals, but lower among younger individuals (as seen in Figure 1). We have now rectified the error.

Introduction and methods
The introduction is compact and to the point.
However, the method section is hard to read without reading additional information from the protocol paper, the trial registry, or the protocol, and it should not be like that. The flow chart is confusing, because there is follow up for a subgroup of 30-60-90 days according to the trial registry, and we have to assume these all complied? It is also not clear what the sample size is for this subgroup in each arm. It is also not clear if the eligible persons for RFDA are for the same 140 meters as for the RFTAT group. Was there supervision of the DHAP in the RFDA arm? (This information is in the protocol paper but could be added in a few words).

We have clarified that a modified DOTS procedure (first and last dose only) was used for DHAP and AL administration. We have updated the CONSORT statement to be the one for cluster-randomized controlled trials, which will hopefully reduce some of the confusion. We have also added all deviations from the protocol to a new sub-section on “Evaluation Study Design” in the methods. Finally, we have added the additional data on the 30- and 90- day follow-ups in the manuscript.

The second secondary outcome is not presented in this paper (PCR parasite prevalence among individuals participating at 0, 30 and 90 days following a reactive research response for a period of 24 months)?

We have now added this information in as a supplementary table (S9 Table) and conducted a Fishers exact test which was suggestive of the RFTAT control arm being inferior to the RFDA intervention arm at Day 30. However, by day 90 there was no difference between the two arms. It is worth noting that the total number of reinfections at day 30 and 90 were very small and there was a high loss to follow-up of participants potentially biasing our sample set.

The initial outcome was among children <5 years of age, but this was apparently changed to <15 years of age, with a referral to the protocol paper. I screened that paper but still can't find the reason why, may have missed it. The protocol also talks about <15 years of age, but I can't find a list with changes to the protocol where this is explained.
Why were samples from infants (<1 year old) not included?

We have now clarified in the methods that we revised the inclusion criteria to children <15 years of age to increase sample size in the cross-sectional survey and allow the <5 to be compared to the >5. We excluded children < 1 years of age to avoid complications from maternal antibodies.

I don't really understand how the DND model was used when there was just one timepoint at the end, and not the usual two surveys comparing beginning and end, and intervention. Was child age used as a proxy for time? Given that the intervention lasted two years, were other age cut-offs tried out?

As the reviewer has guessed we used child age as a proxy for time. We have clarified this in the methods section. We *a priori* stratified as <5 and 5-15 and have used this as our main outcome. We did perform a sensitivity analysis by changing the age threshold and have included this too.

Results
The reasons for the persons not receiving the allocated intervention in the RFDA arm could be added as a footnote to Figure 1. The flowchart gives the impression of a hurried job, with these instructional texts "give reasons" still in, as if it is not yet finished. If there is 0, this instructional text could be removed.

We have revised the flow chart to the cluster randomized trial and corrected these omissions.

Table 1: how can there be more than one RDC response to a confirmed malaria case?

This was due to CHW’s responding incorrectly to clinical cases of malaria e.g. an individual presenting with fever, but RDT negative. These responses happened early in the trial and were later rectified.

Can there be a characteristics table in the supplement for the children, so readers can eyeball if the results by arm, especially for the factors used for adjustment?

We have now included a characteristics/ demographics table in the supplementary data (S11 Table).

"Adjusted model included factors of wealth quintile, head of household education, household electricity access, house had open eaves or not, whether the child slept under an insecticide-treated mosquito net the previous night and for the short term antigens age in years."
What is short term antigens age in years? Is this used here as an indicator of malaria transmission in the area?

We have reworded the quoted section. For clarity we employed two models for the long and short term antigens. While similar there were two differences. Firstly, the long-term antigen model includes an arm by age interaction term while the short-term antigen model does not. Secondly, the short-term antigen model includes age in years while the long-term antigen model does not.

Line 12-13: Positivity for AMA-1 or MSP1-19 alone were ~11% while GLURP-R2 alone was 27% (S6 Table). Comment: Is this table S6 or table S1? In S1, instead of showing number of positive and negative results, can be shown positive (percentage) and total sample in columns? This makes it easier to verify.

Thank you for identifying this misattribution - we have changed the reference for S6 to S1. We have also reformatted the table to include antigen positivity.

I could not find the captions and legends for the graphs belonging to the main text, and there were these missing referrals (e.g. "Error! Reference source not found.,") which made it hard to puzzle the results figures together.

Apologies for this, the captions are all in the main text and the reference errors have now been rectified.

"although the same AE data collection system was in place". What was the AE data collection system? This was the adherence visit at day 3? Please add to methods so it makes more sense.

We have added the following statement to the methods regarding adverse events: “During the day 3 visit, adverse events were also recorded by the CHW.”

Discussion
In my opinion, the following section could be moved to the methods section, to pre-empt questions:
"In the first stage, we leveraged long-lasting IgG responses (> 5-7 years) into a difference-in-differences (DID) (27) comparison, that examines the difference between pre- and post-intervention measures (the differences) between intervention and control groups (the difference). For the CoRE study this means comparing how differently the two arms of the trial changed over time. Typically, a DID approach requires a pre-intervention measure, but as we applied it here, we used seropositivity in children aged 5-15 as a measure of pre-intervention exposure."
I am not sure what the two stages are referring to in the discussion: two analyses using serological outcomes were performed according to the methods section. Stage suggests that you used one model as a precursor for the next model, but as far as I understand that was not the case. However, if I understand it wrong, I would appreciate if you could improve the language so I can understand it in one go.

We have modified the text to clarify that the two analyses were independent but supported each other. We have moved some of this text to the methods for clarity but have retained the rest as part of the discussion.

Supplement
It is time consuming to click through all these separate supplemental tables and figures to be able to see them. Can they be combined in one supportive PDF file? E.g., I wanted to check if there was a characteristics table of the participants in the surveys at the end of the intervention. That will also help with the figures, because now they are presented without text underneath, and it is cumbersome to find out what they represent.

Apologies for this – we were following journal requirements.

Dataset
Note that with the dataset only the unadjusted analyses can be verified, not the adjusted. I could replicate the AMA-results of Table S1.

We have included all the necessary data in the supplemental data.
